# Ghrelin reverses ductular reaction and hepatic fibrosis in a rodent model of cholestasis

**DOI:** 10.1038/s41598-020-72681-5

**Published:** 2020-09-29

**Authors:** Anca D. Petrescu, Stephanie Grant, Elaina Williams, Gabriel Frampton, Evan H. Reinhart, Amy Nguyen, Suyeon An, Matthew McMillin, Sharon DeMorrow

**Affiliations:** 1 Central Texas Veterans Health Care System, Temple, TX 76504 USA; 2 Division of Pharmacology and Toxicology, College of Pharmacy, The University of Texas at Austin, Austin, TX 78712 USA; 3 Department of Internal Medicine, Dell Medical School, The University of Texas at Austin, Austin, TX 78701 USA; 4 Department of Internal Medicine, Baylor Scott & White Health, Temple, TX 76502 USA; 5 University of Mary Hardin-Baylor, Belton, TX 76513 USA

**Keywords:** Liver fibrosis, Molecular biology

## Abstract

The orexigenic peptide ghrelin (Ghr) stimulates hunger signals in the hypothalamus via growth hormone secretagogue receptor (GHS-R1a). Gastric Ghr is synthetized as a preprohormone which is proteolytically cleaved, and acylated by a membrane-bound acyl transferase (MBOAT). Circulating Ghr is reduced in cholestatic injuries, however Ghr’s role in cholestasis is poorly understood. We investigated Ghr’s effects on biliary hyperplasia and hepatic fibrosis in Mdr2-knockout (Mdr2KO) mice, a recognized model of cholestasis. Serum, stomach and liver were collected from Mdr2KO and FVBN control mice treated with Ghr, des-octanoyl-ghrelin (DG) or vehicle. Mdr2KO mice had lower expression of Ghr and MBOAT in the stomach, and lower levels of circulating Ghr compared to WT-controls. Treatment of Mdr2KO mice with Ghr improved plasma transaminases, reduced biliary and fibrosis markers. In the liver, GHS-R1a mRNA was expressed predominantly in cholangiocytes. Ghr but not DG, decreased cell proliferation via AMPK activation in cholangiocytes in vitro. AMPK inhibitors prevented Ghr-induced FOXO1 nuclear translocation and negative regulation of cell proliferation. Ghr treatment reduced ductular reaction and hepatic fibrosis in Mdr2KO mice, regulating cholangiocyte proliferation via GHS-R1a, a G-protein coupled receptor which causes increased intracellular Ca^2+^ and activation of AMPK and FOXO1, maintaining a low rate of cholangiocyte proliferation.

## Introduction

Ghrelin (Ghr) is a gastric orexigenic peptide with multiple roles in physiology, including food intake and appetite regulation by gut-brain signaling pathways, as well as metabolism functions related to energy homeostasis^1–4^. Ghr is synthetized predominantly in the stomach and gastrointestinal tract as a preprohormone which is proteolytically processed to a 28-amino acid peptide, and then acylated at serine 3 residue by a membrane-bound acyl transferase (MBOAT)^5^. Both des-acylated and octanoyl-Ghr are secreted into the systemic circulation during fasting, but only octanoyl-Ghr binds to and activates growth hormone secretagogue receptors type 1a (GHS-R1a), located predominantly in the hypothalamic neurons that regulate hunger and metabolism^6–9^. GHS-R1a is a G-protein-coupled receptor expressed in many tissues including the hypothalamus, pituitary gland, adrenals, thyroid, pancreas, and liver^6^. The expression of GHS-R1a mRNA increases up to eightfold in the hypothalamus during fasting, enhancing Ghr-mediated stimulation of neuropeptide Y (NPY) and agouti-related peptide (AgRP) neurons, with roles in inducing appetite^9,10^.

In the serum of healthy human subjects, two forms of the peptide have been identified, i.e. the acylated Ghr with a half-life of approximately 10 min, and the unacylated or des-ghrelin (DG), which is more stable, having a half-life of 35 min^11^. Systemic Ghr and DG have been reported to be in a ratio of 1/4 to 1/9 respectively in humans, and 1/13 in the mouse^11,12^. Acylation of DG to Ghr has been demonstrated to occur primarily in the stomach by MBOAT also known as Ghr O-acyl transferase or GOAT^13,14^. However, the process of Ghr deacylation is still poorly understood, though it has been suggested that it could take place in the liver since Ghr was found in significant amounts in hepatic tissue^11,15^. Other studies suggest that Ghr is deacylated by serum butyrylcholinesterase^16^, and both Ghr and DG undergo renal clearance following the typical clearance pathway for small peptides^17^.

Interestingly, Ghr was found to be dysregulated in patients with liver injuries^18–21^. Fasting Ghr levels were decreased in adults with cirrhosis, while DG was not changed or increased as compared to healthy volunteers^18^. In Child C cirrhosis patients though, Ghr levels were unchanged or elevated, independent of the etiology of liver disease^19^. In patients with primary biliary sclerosis (PBC), serum Ghr levels were decreased compared to the control groups^22^. A study on the influence of polymorphism in Ghr gene (*GHRL*) on hepatic fibrosis in patients with chronic hepatitis C, found that certain mutations in *GHRL* were associated with more severe liver fibrosis compared to wild type *GHRL*^23^. Only a few publications describe the effects of exogenously administered Ghr on the liver in animal models of hepatic injuries, suggesting that Ghr may have antioxidant and anti-inflammatory outcomes protecting against liver fibrosis due to biliary obstruction^23,24^.

In the present study, we used the Mdr2-knockout (Mdr2KO) mouse model of cholestasis to assess the effects of Ghr on biliary hyperplasia and hepatic fibrosis. In Mdr2KO mice, the ablation of *ABCB4* gene encoding for the multidrug resistance protein 2 (Mdr2), a membrane protein with flippase activity and a role in the hepato-canalicular transport of phospholipids, results in bile retention in the liver and cholestasis-induced hepatic fibrosis^25–27^. Mdr2 is the rodent homologue of human Mdr3 protein, an ATP-binding cassette transporter with an important role in normal bile secretion^28^. Several clinical studies demonstrated that genetic mutations in *ABCC4* gene encoding for Mdr3 were associated with symptomatic intrahepatic cholelithiasis, familial hepatic cholestasis and cirrhosis^29,30^. Therefore, a large number of mechanistic studies of potential therapeutic drugs for treating symptoms of cholestasis-related injuries of the liver have been performed on Mdr2KO mice^31–35^. In the study described herein, we assessed Ghr levels in serum, stomach and liver of cholestatic versus normal mice, as well as how exogenous administration of Ghr and DG influence the expression of fibrogenic genes in vivo. Using cholangiocytes in vitro, we also dissected the molecular mechanism underlining Ghr-mediated regulation of cholangiocyte proliferation.

## Results

The mice used in our experiments were genotyped, and it was confirmed that FVBN mice were *Mdr2*^+/+^, while Mdr2KO mice were *Mdr2*^-/-^ (Suppl Fig. 1).

**Figure 1:**
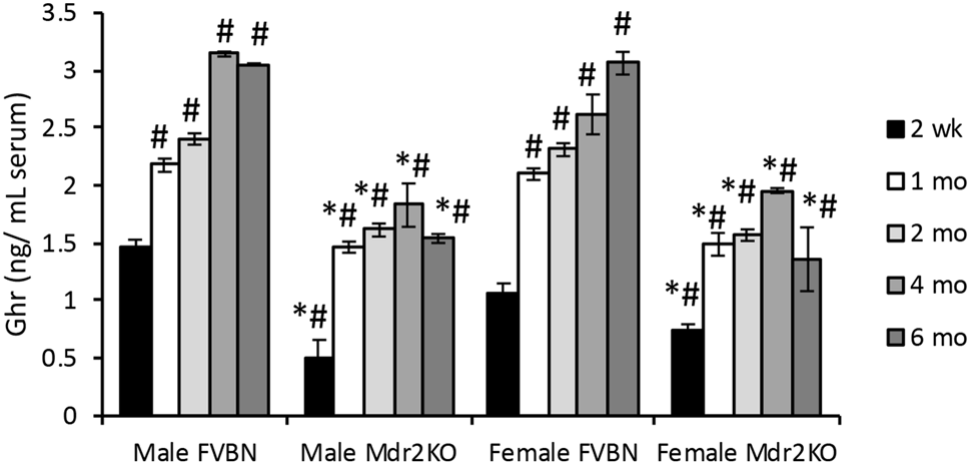
Time-course of serum Ghr in Mdr2KO mice vs FVBN controls. Male and female mice from two weeks to six months old were tested for circulating Ghr by EIA. Number of animals, 4; number of serum sample replicates in ELISA test, 3; p < 0.05. *, Mdr2KO vs FVBN mice. #, all mice older than two weeks vs two week old, male FVBN mice.

### Time-course of serum Ghr in Mdr2KO mice as compared to FVBN control mice

Ghr peptide level was measured in serum of male and female Mdr2KO and FVBN mice from 2 weeks to 6 months old (Fig. 1). In FVBN controls, Ghr concentration increased gradually with age until four months, when it reached a plateau. In Mdr2KO mice, there was a significant increase in circulating Ghr in mice from two weeks up to one month, but no further enhancement was detected in later age groups. Interestingly, at each timepoint tested, the level of serum Ghr was lower in Mdr2KO mice as compared to FVBN mice.

### Expression of Ghr and MBOAT in the stomach of Mdr2KO and FVBN control mice

**Figure 2:**
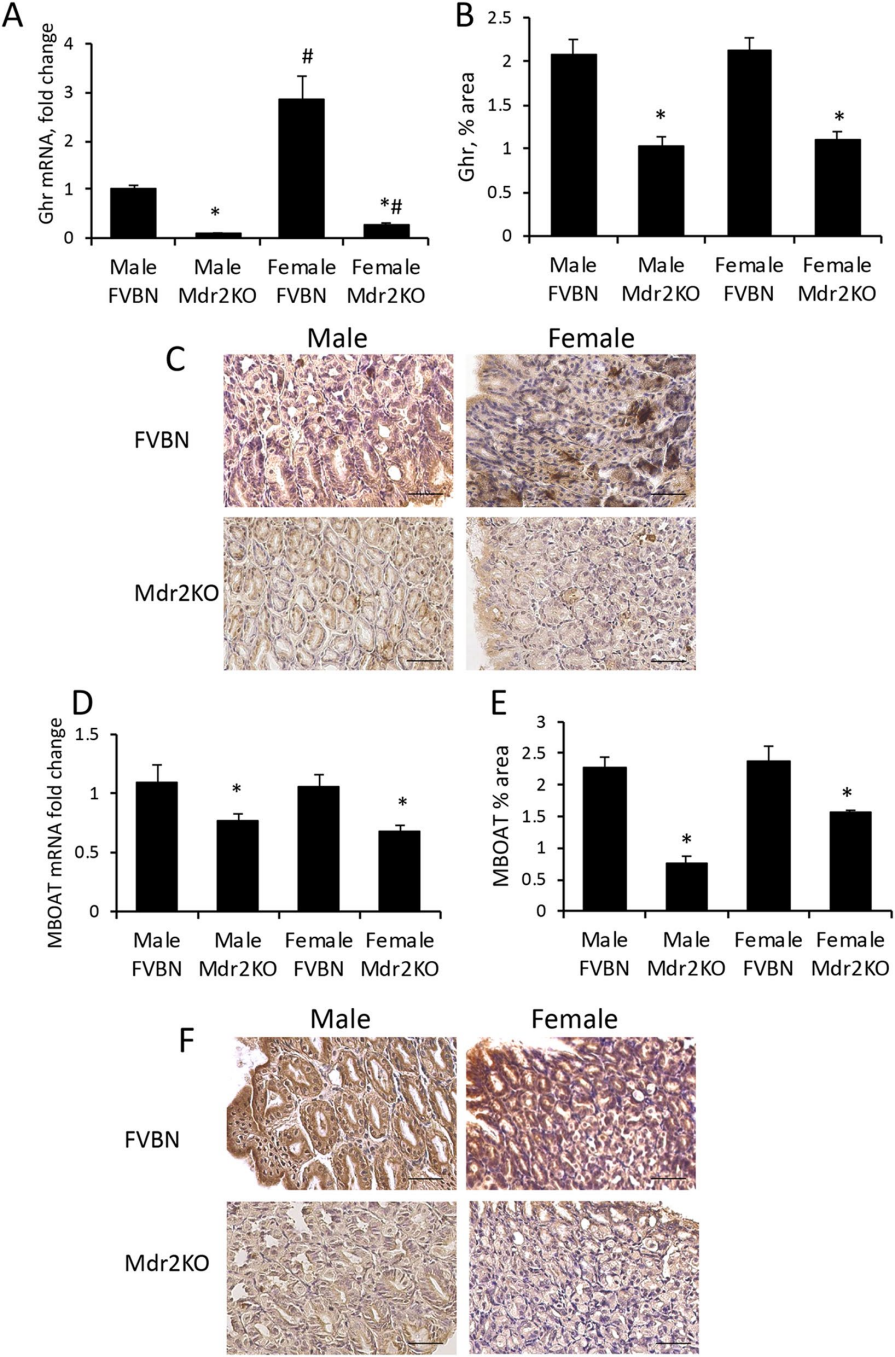
Gastric Ghr and MBOAT expression in Mdr2KO vs FVBN mice. Stomach tissue from 2 month old male and female Mdr2KO and FVBN mice was tested for RNA and protein expression of Ghr and MBOAT, as described in “Methods” section. **(A)** Ghr mRNA expression was assessed by RT-qPCR. **(B)** Quantification of Ghr by analysis of IHC images as shown in **(C)**. **(D)** MBOAT mRNA as assessed by RT-qPCR. **(E)** Quantification of MBOAT by image analysis of IHC images. **(D)** representative images of MBOAT IHC. Number of animals, 4. Number of sample replicates for RT-qPCR, 3. For IHC: 10 images at × 20 magnification for each scanned IHC liver section were taken and analyzed using Image J software, as described in the Methods section. p < 0.05. *, Mdr2KO vs FVBN mice. #, Female vs male.

We assessed Ghr expression in the stomach of Mdr2KO and FVBN mice, since Ghr is mainly produced in this part of the gastrointestinal system (Fig. 2A–C). Ghr mRNA level was significantly lower in cholestatic mice than in normal controls (Fig. 2A). At the translational level, Ghr peptide was also decreased in Mdr2KO mice as compared to FVBN controls (Fig. 2B,C). Similarly, the enzyme which activates Ghr by acylation, MBOAT, was found to be less abundant in Mdr2KO mice over FVBN controls (Fig. 2D–F). There were no significant gender-related differences in Ghr and MBOAT gastric expression.

### Cellular distribution of GHS-R1a and GHr in the liver of Mdr2KO and FVBN control mice

**Figure 3:**
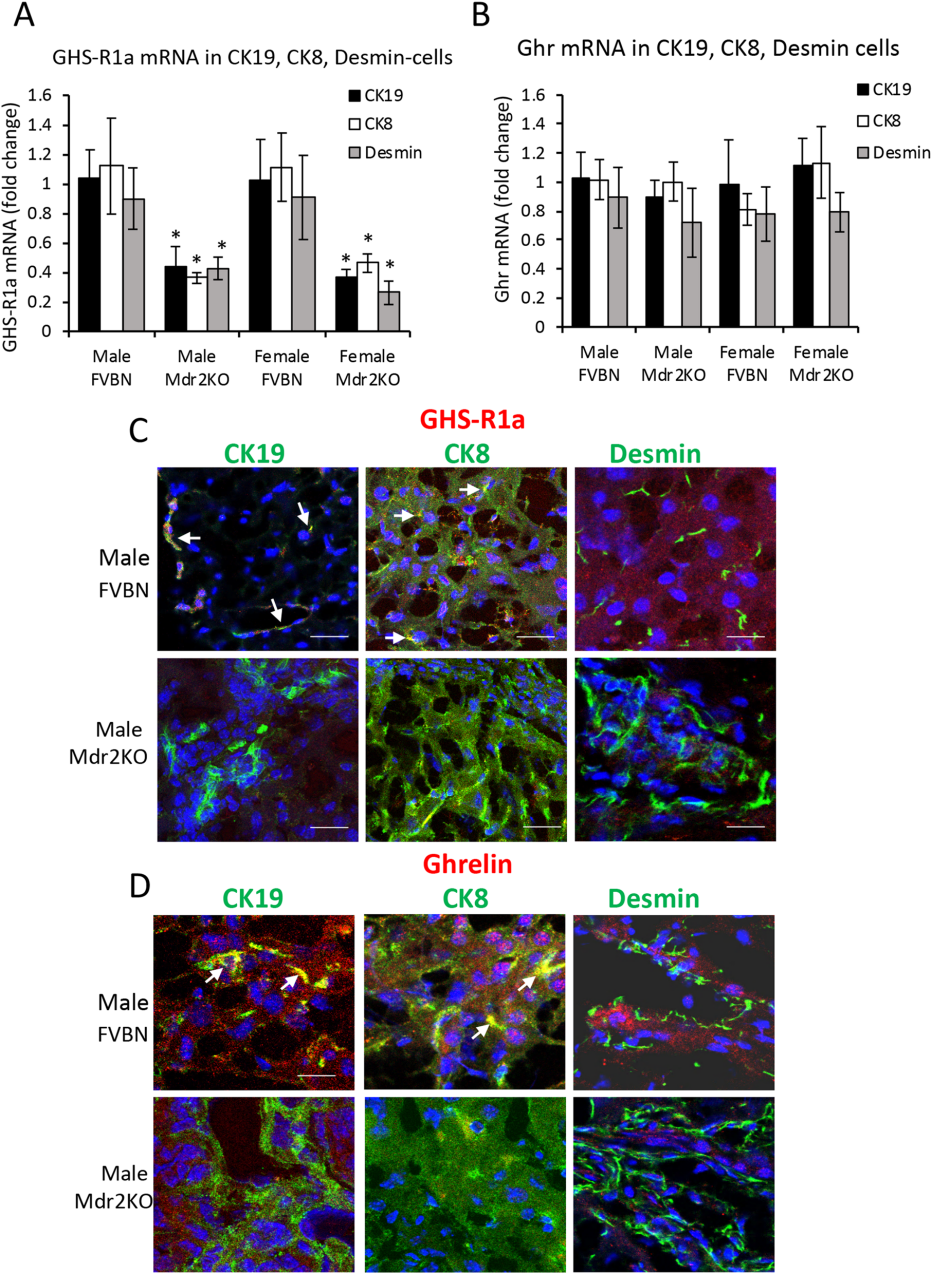
Expression of GHS-R1a and Ghr in liver cells of Mdr2KO vs FVBN mice. **(A, B)** Liver frozen sections from Mdr2KO and FVBN mice, were immunolabeled for CK19 (cholangiocytes), CK7 (hepatocytes) and desmin (HSC). The labeled cells were dissected by laser capture microdissection (LCM) and used for RNA isolation and quantitative determination of GHS-R1a and Ghr mRNAs (fold change relative to GAPDH gene control). Number of animals for each type of treatment, 4. Number of sample replicates for RT-qPCR assay, 3. p < 0.05. *, Mdr2KO vs FVBN mice. C, D At protein level, GHS-R1a and Ghr were detected in same cell types by confocal microscopy colocalization with CK19, CK8 and desmin. **(C)** Representative images of GHS-R1a (red) colocalization with cholangiocytes, hepatocytes and HSC (green). **(D)** Representative images of Ghr (red) with cholangiocytes, hepatocytes and HSC (green). The pixels with both green and red fluorescence signals are shown in yellow (also pointed out by arrows). Cell nuclei were stained with DAPI (blue).

To investigate the expression of GHS-R1a and Ghr in isolated cholangiocytes, hepatocytes and hepatic stellate cells (HSC) from livers of Mdr2KO mice vs FVBN controls, we used laser capture microdissection (LCM). For optimal precision, cholangiocytes, hepatocytes and HSC were labeled for two different markers, i.e. cytokeratin (CK) 19 and CK7 for cholangiocytes, CK8 and albumin (Alb) for hepatocytes, desmin and alpha smooth muscle (αSMA) for HSC (Fig. 3A,B, Suppl Fig. 2). While desmin is expressed in all HSC including quiescent, activated and inactivated cells, αSMA is expressed only in activated HSC. GHS-R1a mRNA was more abundant (ΔCt 3–4) as compared to Ghr (ΔCt 8–10), in all groups of mice. In Mdr2KO mice, GHS-R1a mRNA was significantly lower compared to FVBN controls in cholangiocytes, hepatocytes and HSC (Fig. 3A, Suppl Fig. 2A). Ghr mRNA was detected in all tested cell types at the same level in FVBN and Mdr2KO mice, with no gender-related differences (Suppl Fig. 3B). However, the results indicated that Ghr mRNA was significantly lower in αSMA-expressing HSC (Suppl Fig. 2B) than in desmin-expressing HSC (Fig. 3B), suggesting that HSC activation and change from fibroblast to myofibroblast phenotype is associated with a decrease in Ghr mRNA.

The expression of GHS-R1a and Ghr at protein level in different types of hepatic cells was assessed by confocal microscopy colocalization of GHS-R1a or Ghr with CK19 in cholangiocytes, CK8 in hepatocytes and desmin in HSC (Fig. 3C,D for male mice, and Suppl Fig. 3 for female mice). Ghr peptide (Fig. 3D) as well as GHS-R1a (Fig. 3C) were detected in the liver of FVBN mice, colocalizing with CK19 and CK8, and less so with desmin. The level of Ghr and its receptor in the liver of Mdr2KO mice was reduced as compared to FVBN controls. These results indicate that most of Ghr in the liver comes from the systemic Ghr and acts upon GHS-R1a receptors on cholangiocytes and hepatocytes.

### Ghr treatment of Mdr2KO mice attenuates serum biomarkers of liver disease

**Figure 4:**
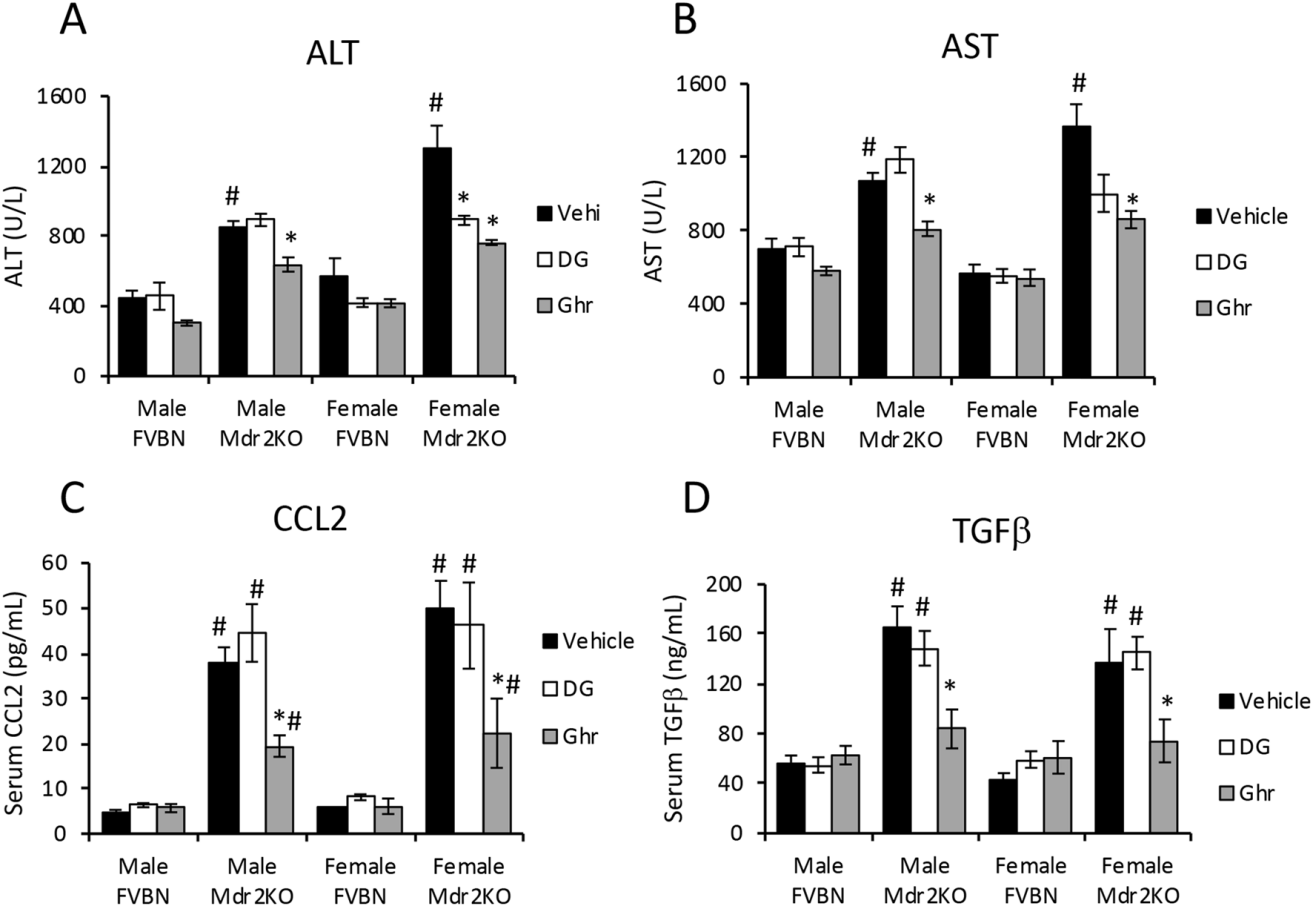
Ghr reduces serum biomarkers of liver injury and inflammation in Mdr2KO mice. Liver transaminases ALT **(A)** and AST **(B)** were assessed in serum from Mdr2KO and FVBN mice when treated with vehicle, DG or Ghr. ALT and AST were quantified using an IDEXX analyzer system, as described under Methods. The proinflammatory cytokine CCL2 and the pro-fibrogenic growth factor TGFβ1 were assayed using ELISA kits. Number of animals of each treatment group, 4, and number of sample replicates for IDEXX and ELISA, 3. p < 0.05. *, Ghr or DG vs vehicle. # Mdr2KO vs FVBN mice.

The effects of exogenous Ghr and its des-acylated form, DG, on the serum liver enzymes, when administered to cholestatic vs control mice, were assessed. While DG had no effect on alanine aminotransferase (ALT) and aspartate aminotransferase (AST), Ghr induced a significant reduction in serum levels of these liver enzymes in Mdr2KO mice (Fig. 4A,B). Serum bilirubin and albumin were slightly changed in Mdr2KO mice compared to FVBN control mice treated with vehicle, and the treatment with DG or Ghr corrected these changes (Suppl. Table 1). The inflammatory chemokine CCL2 (C–C chemokine 2), also known as monocyte chemotactic/chemoattractant protein 1 (MCP1) was found to be more than eightfold-increased in serum of male and female Mdr2KO mice compared to FVBN controls (Fig. 4C). Administration of DG did not change the high plasma level of CCL2, however treatment with Ghr significantly reduced CCL2 in Mdr2KO mice by twofold (Fig. 4C). Serum tumor growth factor beta (TGFβ), the product of the most active profibrogenic gene in hepatic cholestasis, was also tested and found to be increased 3 to 4- fold in both male and female Mdr2KO mice compared to FVBN control counterparts (Fig. 4D). Treating the Mdr2KO mice with DG had no effect on TGFβ level, while Ghr reduced approximately by half the plasma TGFβ concentration (Fig. 4D). In summary, Ghr, but not DG, was effective in reducing the serum levels of ALT, AST, CCL2 and TGFβ which were remarkably increased in Mdr2KO mice as compared to FVBN controls.

### Ghr decreases intrahepatic bile duct mass (IBDM) and cholangiocyte proliferation in Mdr2KO mice

Because no gender-related differences were found in serum levels of major biomarkers of liver disfunction in Mdr2KO mice when treated with vehicle, DG or Ghr, we used only male mice for the subsequent experiments.

**Figure 5:**
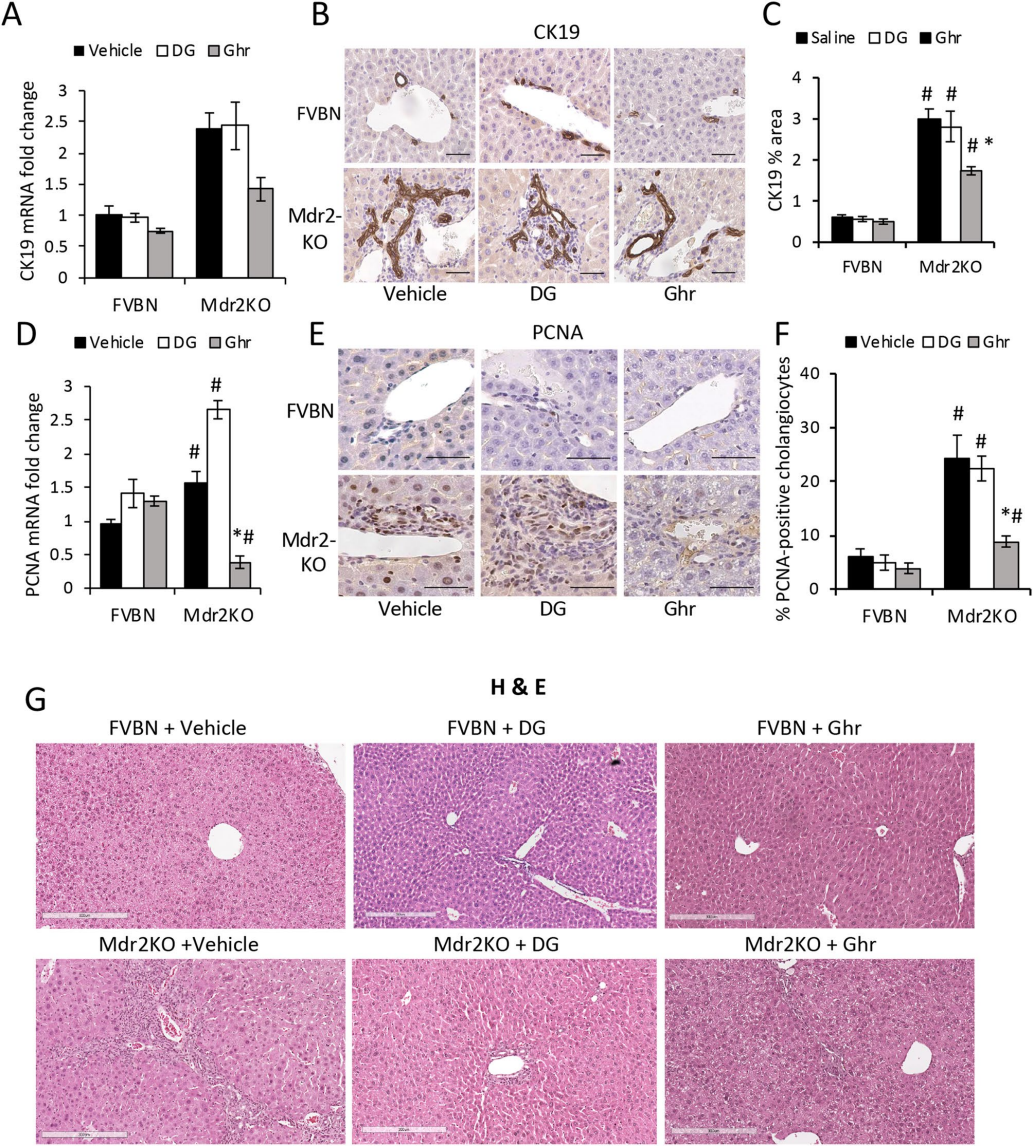
Ghr reduces the IBDM in Mdr2KO mice. Liver samples from male Mdr2KO and FVBN mice treated with vehicle, DG or Ghr, were tested for expression of CK19 mRNA, by RT-qPCR **(A)** and CK protein by IHC **(B, C)**. **(B)** Representative images of CK19 (marker of cholangiocytes) IHC. **(C)** Quantification of CK19 expression by image analysis using ImageJ software. PCNA, a marker of cell proliferation was also tested at RNA **(D)** and protein **(E, F)** level using RT-qPCR and IHC, respectively. **(E)** Representative images of PCNA IHC. **(F)** Quantification of PCNA-expressing cholangiocytes by analysis of IHC images. p < 0.05. * DG or Ghr vs vehicle. #, Mdr2KO vs FVBN mice. Number of animals in each treatment group, 4. Number of sample replicates in RT-qPCR assay, 3. Number of images from each animal in a treatment group, in IHC assay, 10. G, Representative images of H&E staining of liver tissue from male and female FVBN (control) mice and Mdr2KO (cholestatic) mice treated with vehicle, DG or Ghr.

The effects of DG and Ghr on the expression of CK19 in the liver of cholestatic vs control mice was assessed at mRNA and protein level (Fig. 5A–C). Ghr but not DG, caused a significant reduction in the excessively large IBDM of Mdr2KO mice. Proliferating cell nuclear antigen (PCNA) expression was assessed in cholangiocytes of Mdr2KO mice (Fig. 5D–F). Both the mRNA and protein of PCNA were drastically decreased in Mdr2KO mice after treatment with Ghr, but not with DG.

Hematoxylin and eosin (H&E) staining of liver sections from Mdr2KO mice and FVBN controls was used to assess the status of hepatocytes, as well as the size and frequency of biliary mass (Fig. 5G). It can be observed that there were no effects of DG or Ghr on the liver histology in FVBN controls, as expected (top images in Fig. 5G). However, vehicle treated Mdr2KO mice exhibited an increased number of bile ducts, surrounded by thick layers of small cells including string-like shaped HSC. The treatment of Mdr2KO mice with DG had only a small effect on the enlarged biliary mass, however Ghr was very efficient in reducing the size of IBDM. No significant damage was noted in regard to the shape of hepatocytes in Mdr2KO mice, neither steatosis or other histological changes were noted.

These data suggest that exogenously administered Ghr reduces proliferation of cholangiocytes and decreases the size of HSC layers around bile ducts in cholestatic mice.

### Ghr treatment alleviates liver fibrosis in Mdr2KO mice

**Figure 6:**
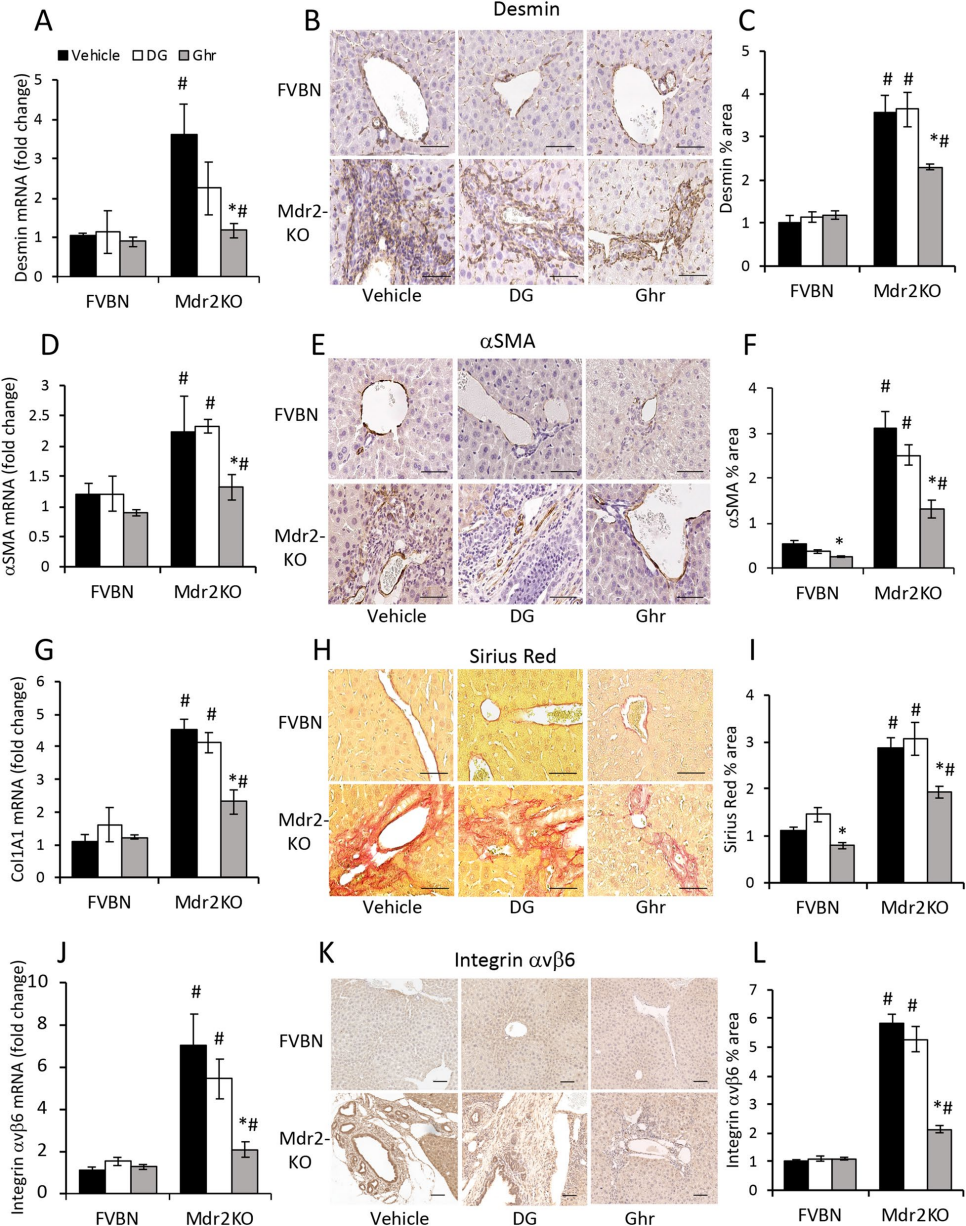
Ghrelin reduces the expression of fibrosis markers in livers of Mdr2KO mice. Liver samples from male FVBN and Mdr2KO mice treated with vehicle, DG or Ghr, were used. Desmin mRNA **(A)** and protein **(B, C)**, αSMA mRNA **(D)** and protein **(E, F)**, markers of HSC proliferation and activation were detected and quantified by RT-qPCR, IHC and image analysis as described under Methods. **(G)** Collagen 1A1 (Col1A1) mRNA was assayed by RT-qPCR. **(H, I)** Collagens I and III were assessed by Sirius Red staining of paraffin sections of liver from FVBN and Mdr2KO mice. **(J)** Integrin αvβ6 mRNA expression was determined by RT-qPCR. **(K)** Representative images of integrin αvβ6 IHC in sections of liver. **(L)** Quantification of αvβ6 protein expression in the liver using analysis of IHC images. Number of animals in each treatment group, 4. Number of sample replicates in RT-qPCR assays, 3. Number of images for each IHC sample, 10. p < 0.05. *, DG or Ghr vs vehicle. #, Mdr2KO vs FVBN mice.

Genes known to be upregulated in cholestasis-induced hepatic fibrosis, including desmin and αSMA markers of HSC, as well as structural proteins of extracellular matrix (ECM) such as collagen types I and III, and integrins, were tested in Mdr2KO mice and FVBN controls treated with vehicle, DG or Ghr (Fig. 6). All the tested genes were downregulated in Ghr-treated Mdr2KO mice while being insignificantly affected by DG. Thus, the expression of both desmin and αSMA was strongly increased in Mdr2KO mice compared to FVBN controls (Fig. 6A–F). The treatment with DG did not change the excessive amount of HSC markers detected in Mdr2KO mice, while Ghr induced a significant decrease of them. Collagen types I and III were assessed using Sirius-Red staining of liver sections, and were found to be produced in excess in Mdr2KO mice treated with vehicle or DG but were reduced in Mdr2KO mice treated with Ghr (Fig. 6G–I). To confirm these results, we also assayed the liver hydroxyproline concentrations (Suppl Fig. 3), and determined that hydroxyproline was excessively expressed in the livers of Mdr2KO mice, and it was not affected by DG, however it was significantly decreased following Ghr treatment (Suppl Fig. 3). The β6 component of integrins was demonstrated to be more than sevenfold increased at mRNA level in the livers of Mdr2KO mice compared to FVBN controls, and it was strongly diminished in Mdr2KO mice treated with Ghr, while DG had only small alleviating effect (Fig. 6J). Moreover, IHC assessment of integrin αvβ6 indicated that the massive increase in this integrin expression within the thick layers of HSC around enlarged bile ducts in Mdr2KO mice was counteracted by treatment with Ghr, while DG had no effect (Fig. 6K–L).

**Figure 7:**
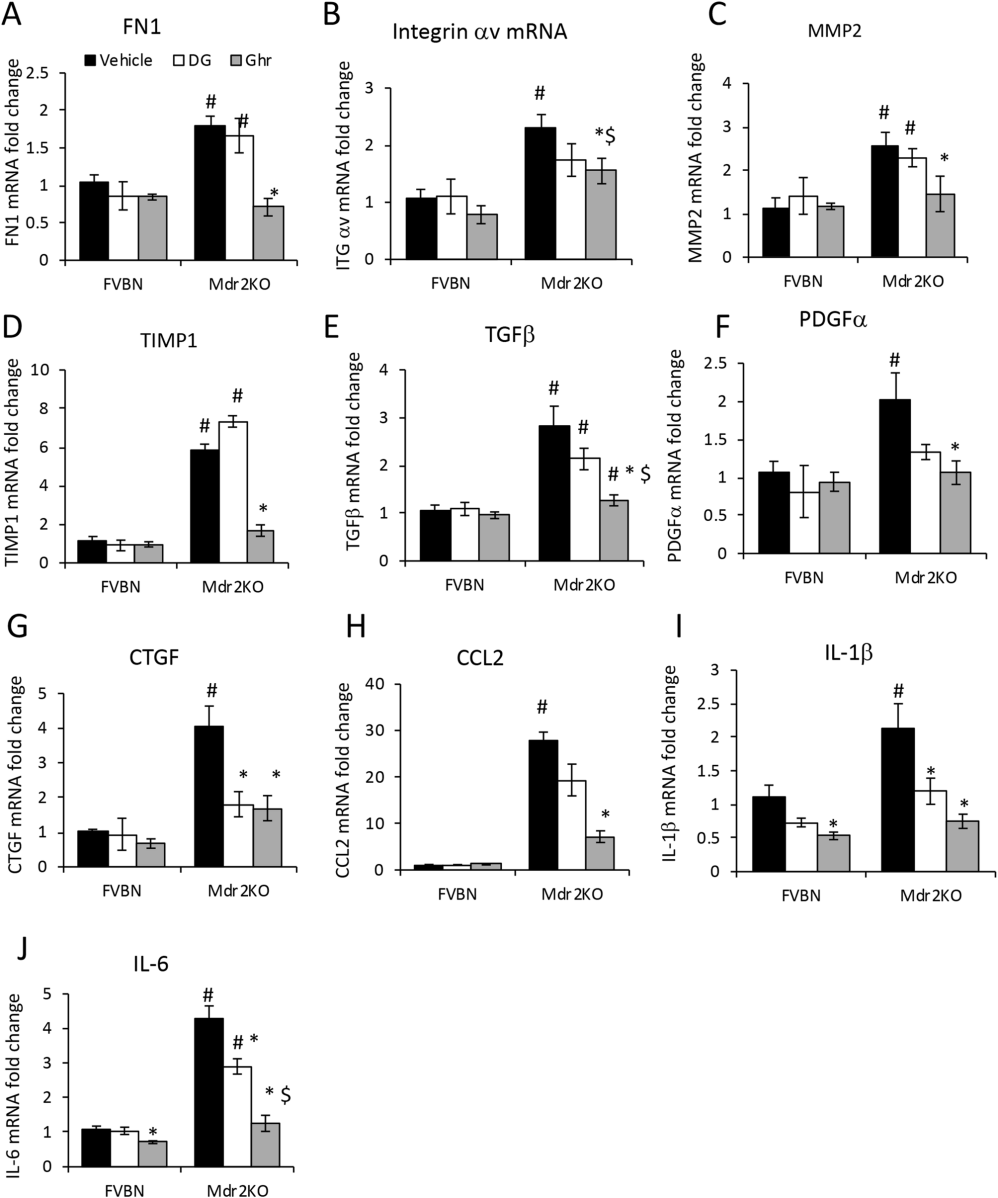
Ghrelin attenuates expression markers of hepatic fibrosis and inflammation in Mdr2KO mice. The expression ECM components FN1 **(A)**, Integrin αv **(B)**, MMP2 **(C)** and TIMP1 **(D)** at RNA level was performed by RT-qPCR in male Mdr2KO and FVBN mice that had been treated with vehicle, DG or Ghr. The expression of profibrogenic genes including TGFβ **(E)**, PDGFα **(F)**, CTGF **(G),** as well as proinflammatory cytokines CCL2 **(H)**, IL-1β **(I)** and IL-6 **(J)**, was also tested by RT-qPCR in the same groups. Number of animals per treatment group, 4. Number of sample replicates in RT-qPCR assay, 3. p < 0.05, *, DG or Ghr vs vehicle. #, Mdr2KO vs FVBN mice.

The expression of additional genes with roles in liver fibrogenesis was tested at mRNA level in Mdr2KO mice treated with vehicle, DG or Ghr, and compared to FVBN controls subjected to similar treatments (Fig. 7). Thus, fibronectin (FN1), integrin component αv, matrix metalloproteinase-2 (MMP2), and tissue inhibitor of MMP-1 (TIMP1) were abnormally increased in Mdr2KO mice treated with vehicle. Ghr but not DG, was effective in reducing mRNA expression of these genes (Fig. 7A–D). Profibrogenic genes such as TGFβ, PDGFα (platelets derived growth factor alpha) and connective tissue growth factor (CTGF) which are major indicators of liver fibrosis, were assayed using qPCR and shown to be significantly increased in Mdr2KO mice compared to FVBN controls (Fig. 7E–G). Interestingly, DG, the less active form of Ghr, had a trend to decrease the expression of these genes, and it had a significant effect on CTGF. Ghr was more effective than DG, drastically lowering the expression of all tested growth factors. Finally, several proinflammatory genes including CCL2, interleukin (IL)-1β, IL-6 were assessed in livers of Mdr2KO mice vs FVBN controls (Fig. 7H–J). CCL2 was the most increased (more than 25-fold), followed by IL-6 (fourfold) and IL-1β (twofold) in Mdr2KO mice compared to FVBN controls. DG did not decrease CCL2 mRNA, but had a significant effect on IL-1β and IL-6, while Ghr reduced the expression of all tested cytokines (Fig. 7H–J).

In summary, major indicators of hepatic fibrosis in Mdr2KO mice including biomarkers of proliferating and activated HSC, ECM structural proteins and modulators, profibrogenic and proinflammatory genes were strongly reduced in mice treated with Ghr. The expressions of fewer genes, namely profibrogenic growth factors and cytokines were also diminished by DG.

### Ghr reduces apoptosis and necrosis in Mdr2KO mouse livers

**Figure 8:**
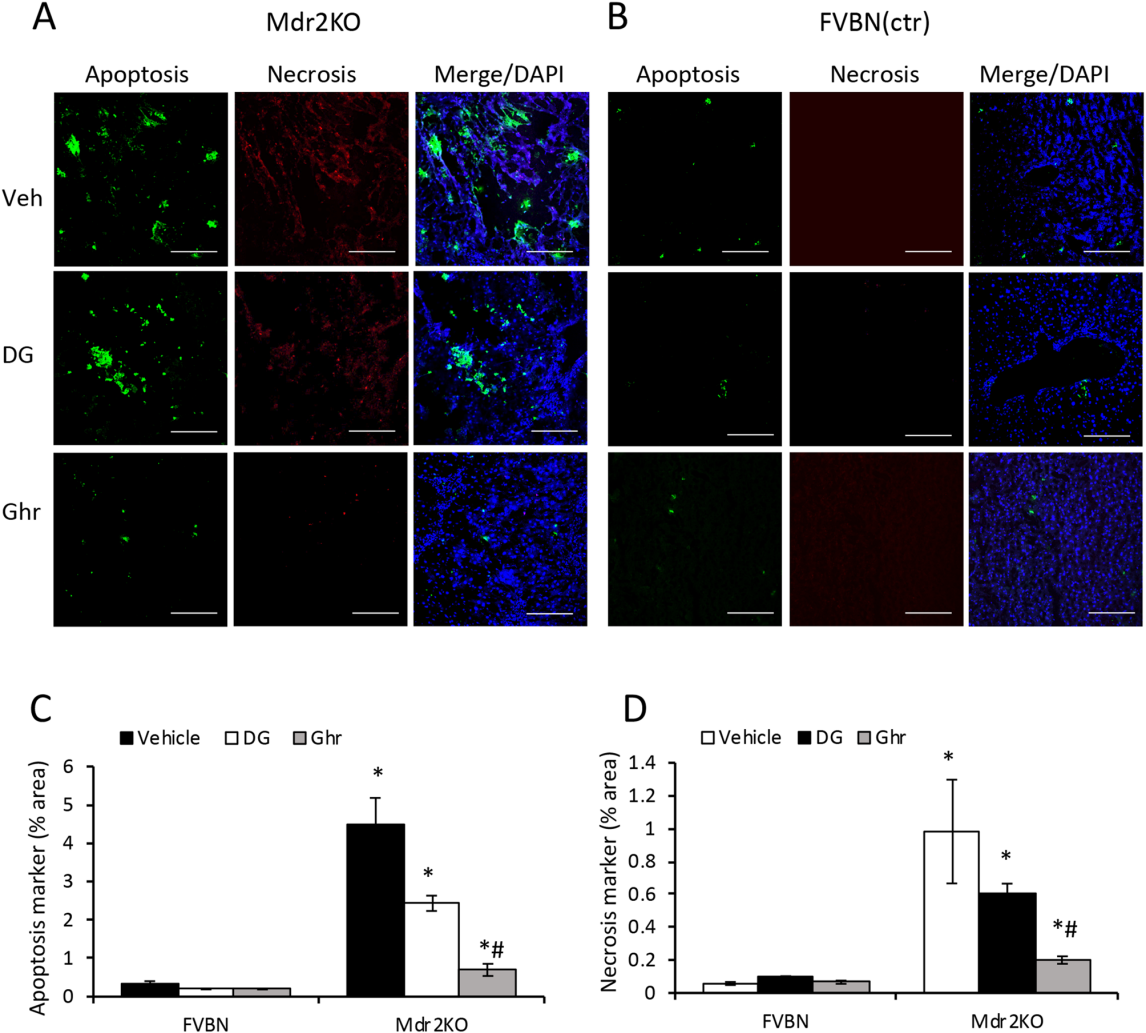
Treatment of Mdr2KO mice with Ghr and DG reduces apoptosis and necrosis markers in the liver. Frozen sections of livers from male Mdr2KO **(A)** and FVBN **(B)** mice treated with vehicle, DG or Ghr, were processed for fluorescence labeling of apoptosis (green) and necrosis (red) markers, while the cell nuclei were stained in blue using DAPI, as described under “Methods”. Panels **(A)** and **(B)** present representative images of each treatment group. **(C, D)** Graphs of quantitative determinations of apoptosis and necrosis, respectively, by image analysis using Image J software. Number of animals in each treatment, 4. Number of images per each sample, 10. p < 0.05, *, DG or Ghr vs vehicle. #, Mdr2KO vs FVBN mice.

To investigate whether DG and Ghr influence apoptosis and necrosis in the Mdr2KO model of cholestasis-induced liver fibrosis, we measured products of these processes using a fluorescence microscopy procedure. Thus, we used a kit for specific staining of phosphatidylserine (PS), a hallmark of apoptosis, and of nuclei of necrosis-damaged cells, and demonstrated that both DG and Ghr significantly decreased apoptosis and necrosis in the liver of Mdr2KO mice (Fig. 8). By image analysis we determined that Ghr was more effective than DG in diminishing apoptosis and necrosis markers.

### Ghr attenuates cholangiocyte proliferation via AMP-activated protein kinase (AMPK) and forkhead box protein O1 (FOXO1) activation pathway

**Figure 9:**
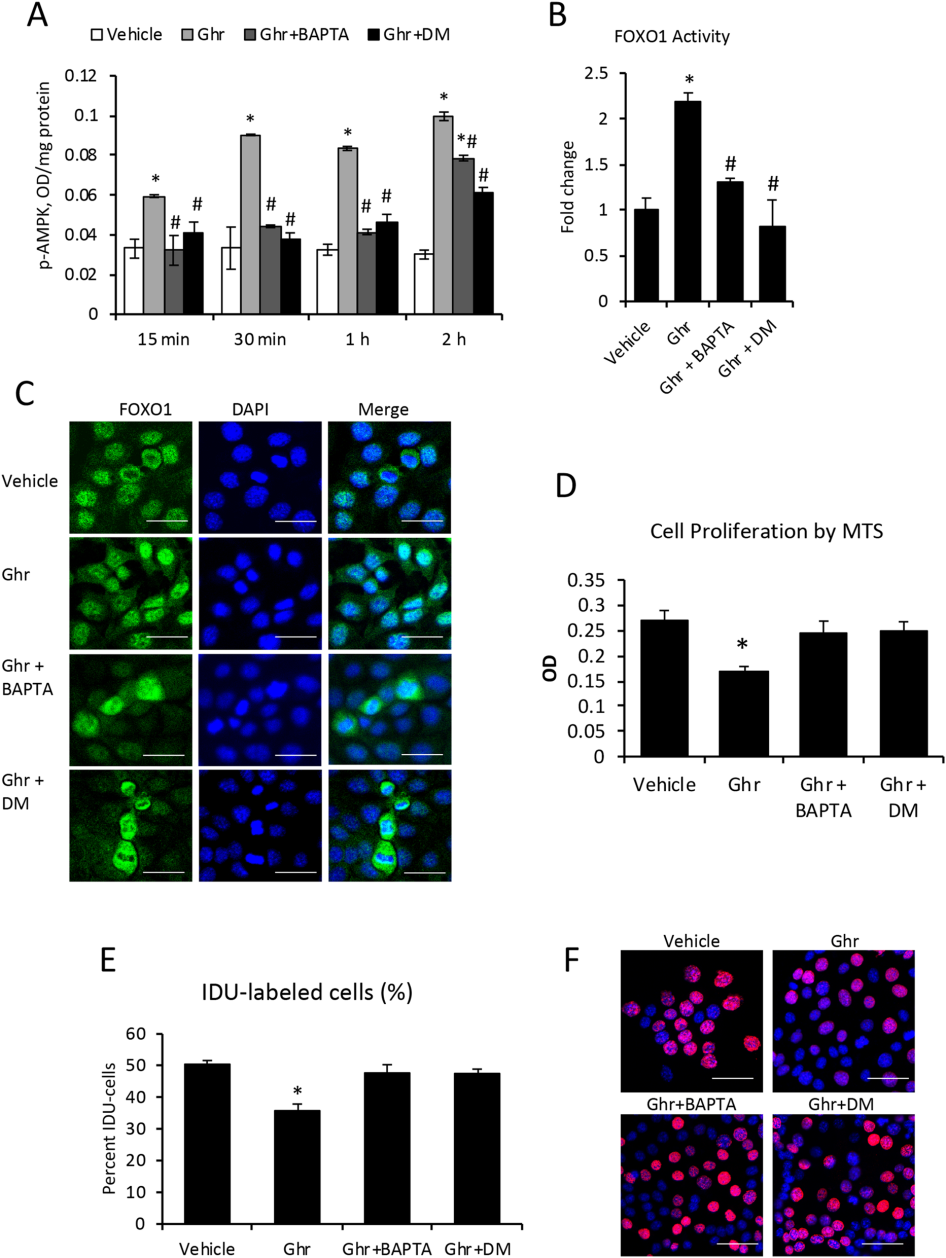
Ghrelin reduces cholangiocyte proliferation by the activation of AMPK-FOXO1 signaling pathway. Mouse cholangiocytes in vitro were treated with vehicle or Ghr in the absence or presence of BAPTA inhibitor of Ca^2+^ mediated activation of calmodulin-dependent kinase kinase or CaMKK), or DM, an inhibitor of AMPK phosphorylation. The p-AMPK was assessed at timepoints from 15 min to 2 h, using ELISA kit as described under “Methods” **(A)**. FOXO1 transactivation **(B)** and nuclear translocation **(C)** were determined 1 h after treatments, using TransAM ELISA kit, and immunofluorescence followed by confocal microscopy, respectively. Cholangiocyte proliferation was assessed using MTS kit **(D)**: the cells were treated with vehicle, Ghr, Ghr + BAPTA or Ghr + DM, and MTS reagent, followed by 4 h incubation and reading of MTS absorbance in a plate reader. A second method to measure cell proliferation was IDU incorporation in nuclei of replicating cells: cholangiocytes were plated one day before treatment, then treated with vehicle, Ghr, Ghr + BAPTA, Ghr + DM for 2 h, followed by fluorescence immunolabeling, confocal microscopy and image analysis. E, quantification of percent IDU-labeled cells for each treatment group. F, Representative images of IDU (red)-labeled nuclei vs DAPI (blue)-stained nuclei of all cells. Number of cell treatment replicates, 3. Number of replicates for ELISA, 3; for MTS, 7; for IDU test, 3. Number of images used for ImageJ analysis, 10 for each treatment sample. p < 0.05. * Ghr vs vehicle.

Activation of AMPK in response to Ghr was tested by measuring phospho-AMPK (p-AMPK) in mouse cholangiocytes in vitro (Fig. 9A). The cells were treated with Ghr in the absence or presence of Ca^2+^ chelator BAPTA or AMPK inhibitor dorso morphine (DM), which are known to block AMPK activation. Phospho-AMPK was quantified at various timepoints up to 2 h using an ELISA kit. Ghrelin induced a significant increase in p-AMPK as early as 15 min after treatment and the effect of Ghr lasted up to several hours. The inhibitors of AMPK phosphorylation and activation, BAPTA and DM, counteracted the effect of Ghr up to two hours.

The AMPK-mediated activation of FOXO1 initiated by Ghr signaling in cholangiocytes was also tested (Fig. 9B). FOXO1 activation in cholangiocytes treated with Ghr only or Ghr plus BAPTA or Ghr plus DM was tested, demonstrating that Ghr induced a two-fold increase in binding of activated FOXO1 to specific DNA response elements. This activation of FOXO1 was not detected in the presence of BAPTA and DM (Fig. 8B), suggesting that FOXO1 activation is dependent on AMPK activation.

It is known that FOXO1 is located inside the nuclei when activated, binding promoters of target genes with roles in cell proliferation control^36,37^. We assessed the nuclear translocation induced by Ghr in cholangiocytes (Fig. 9C). In cells treated with vehicle, FOXO1 was mostly cytoplasmic, detected around nuclei, while after 15 min–2 h of Ghr treatment, FOXO1 was detected inside nuclei in many cells. The inhibitors of AMPK activation, BAPTA and DM, prevented Ghr-induced accumulation of FOXO1 inside nuclei, and even caused upregulation of FOXO1 in the cytoplasm. Some proliferating cells were detected one hour or longer after treatments with BAPTA or DM in addition to Ghr.

To further test the possibility that Ghr affects the proliferation rate of cholangiocytes via AMPK-FOXO1 signaling pathway, we assessed cell proliferation after treatment with Ghr alone or in addition to BAPTA or DM (Fig. 9D). Ghr suppressed cell proliferation while BAPTA and DM prevented this effect (Fig. 9D). To confirm these results, we also measured the incorporation of IDU into mouse cholangiocytes treated with vehicle, Ghr, Ghr plus BAPTA, and Ghr plus DM (Fig. 9E,F). The results suggested that Ghr reduced cell proliferation, however BAPTA and DM attenuated this effect.

Taken together, these data demonstrate that Ghr inhibits cholangiocyte proliferation via a mechanism involving Ca^2+^ and AMPK-mediated nuclear translocation of FOXO1.

### Silencing of GHS-R1a with siRNA in mouse cholangiocytes reverses the effects of Ghr

**Figure 10:**
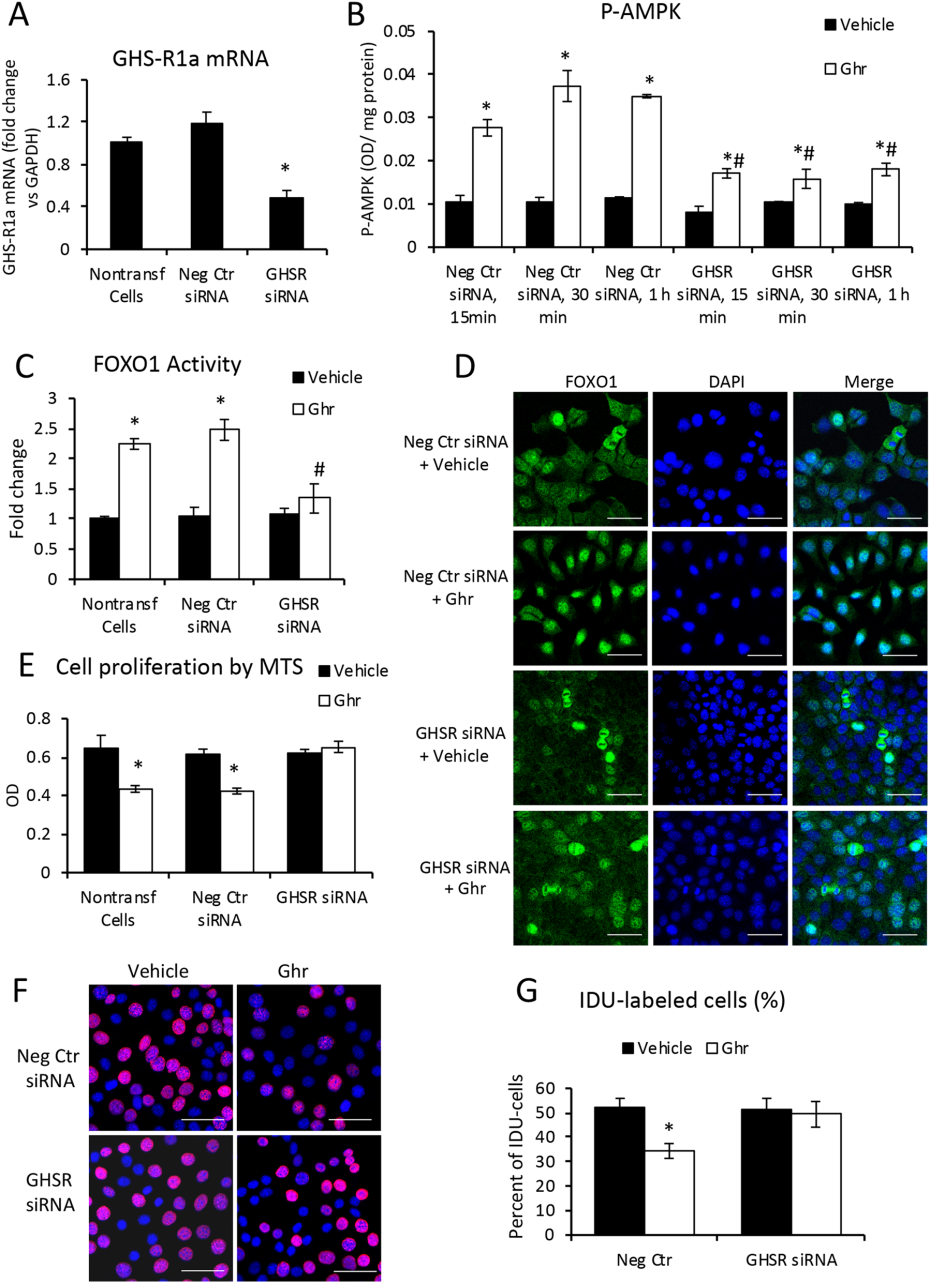
Silencing GHS-R1a receptor in cholangiocytes reduces Ghr-induced signaling pathway through p-AMPK-FOXO1 and cell proliferation. Mouse cholagiocytes were transfected with GHS-R1a-specific siRNA or negative control silencer as described under Methods. The efficiency of siRNAs was checked at 48 h after transfections, by RT-qPCR vs nontransfected and negative control transfected cells **(A)**. A time-course of AMPK phosphorylation in cholangiocytes transfected with siRNA for GHS-R1a or a negative control, was performed using p-AMPK ELISA assay in cells at 15 min to 1 h after treatment with vehicle or Ghr **(B)**. FOXO1 transactivation was assessed using TransAM kit, in cholangiocytes at 48 h after transfection with GHS-Ra1 siRNA or negative control silencer or nontransfected cells, at 1 h after treatment with vehicle or Ghr **(C)**. The intracellular distribution of FOXO1 (green) in nuclei (blue) vs cytoplasm was assessed by immunofluorescence and confocal microscopy of cholangiocytes that were treated with vehicle or Ghr for 1 h after being transfected with negative control of GHS-R1a siRNA 48 h earlier **(D)**. Proliferation of cholangiocytes transfected with GHS-R1a siRNA vs negative control siRNA-transfected or non-transfected cells, were assayed using MTS kit; MTS and vehicle or Ghr were added to cells at the same time, and the absorbance was read 4 h later. Proliferation of cholangiocytes was also assessed using IDU incorporation in nuclei of replicating cells **(F, G)**. Number of treatment sample, 3, and of replicates for RT-qPCT 3; for ELISA, 2; for MTS test, 7; for IDU test, 3. Number of images used for ImageJ analysis, 10 for each treatment sample. p < 0.05. * Ghr vs vehicle.

In order to assess the role of GHS-R1a in signaling FOXO1 nuclear translocation and suppression of cell proliferation rate, we knocked down GHS-R1a mRNA, and confirmed the reduction of GHS-R1a expression (Fig. 10A). Mouse cholangiocytes transfected with GHS-R1a-specific siRNA, were then treated with vehicle or Ghr for 15 to 60 min, and AMPK phosphorylation was measured (Fig. 10B). Similarly, cells transfected with negative control siRNA were used as controls. P-AMPK induced by Ghr, was reduced in GHS-R-siRNA transfected cells compared to cells transfected with negative control siRNA, suggesting that Ghr’s effect is dependent of GHS-R1a. FOXO1 transactivation measurements indicated that GHS-R1a knockdown impaired Ghr-induced FOXO1 transactivation (Fig. 10C). These data were confirmed by the immunofluorescence colocalization of FOXO1 with nuclei in cholangiocytes transfected with GHS-R1a-siRNA vs negative control siRNA (Fig. 10D). Moreover, the assessment of Ghr-induced reduction of cell proliferation rate was performed by two procedures: one measuring the percent of live cells (Fig. 10E), and the other by measuring the incorporation of IDU into newly synthetized DNA during cell replications (Fig. 10F,G). Both procedures indicated that knockdown of GHS-R1a reduced the ability of Ghr to stimulate cholangiocyte proliferation.

## Discussion

Since its discovery, Ghr has been largely investigated as an endogenous gastric orexigenic peptide with high affinity for GHS-R1a^6,10,38^. Ghrelin’s functions related to gut-brain signaling^2,3,39^, or the control of glucose metabolism and body weight^7,8,40,41^ are well-known; however, Ghr’s role in the cholestatic liver is poorly understood. In the present study using Mdr2KO mice as a model of biliary cholestasis-induced hepatic fibrosis, we investigated endogenous Ghr levels in chronic cholestasis, as well as the effects of exogenously administered Ghr and its less active form DG on biliary and hepatic pathology. Serum levels of total Ghr in Mdr2KO mice were lower compared to FVBN controls in mice from two weeks to six months of age. We further assessed the expression of Ghr and MBOAT, the acylation enzyme with role in DG activation, in 2-month old mice, since at this age the Mdr2KO mice exhibit cholestasis and hepatic fibrosis. A much lower expression of Ghr and MBOAT was detected in the stomach of cholestatic mice compared to controls. At mRNA level, the expression of gastric DG was eight- to tenfold lower in Mdr2KO mice than in FVBN control mice, demonstrating that hepatobiliary dysfunction resulted in strong downregulation of *GHRL* gene expression in the stomach, the main source of systemic DG and Ghr. These results indicate that during cholestasis, the gastric biosynthesis and activation of DG to fully active Ghr, are drastically impaired, further aggravating the liver injury.

It has been previously reported that Ghr can promote cell survival and inhibit apoptosis, having therapeutic effects on myocardial, intestinal and pancreatic injuries^42–45^. In our study we demonstrate that Ghr attenuates liver cell apoptosis in chronic cholestasis, since treatment of Mdr2KO mice with Ghr caused a significant reduction of apoptosis markers. The less active form of Ghr, DG was also beneficial in attenuating hepatic apoptosis, however its effect was lower than that of Ghr.

The possibility of Ghr being a hepatoprotective factor was investigated in only a few previous studies^23,24,46^. Iseri et al. (2008) demonstrated that exogenous Ghr had anti-inflammatory and antioxidant effects in BDL rats, and proposed a potential therapeutic value of Ghr in protecting against hepatic fibrosis and oxidative injury due to biliary obstruction^24^. Later on, Moreno et al. (2010) studied the effect of recombinant Ghr on acute, carbon tetrachloride-induced liver injury, demonstrating that markers of hepatic fibrosis were increased in *GHRL* null mice as compared to wild type mice^23^. A more recent study proposed a role of Ghr in protecting against liver injury via nitric oxide release^46^. In the present study, we investigated the expression of Ghr and its receptor GHS-R1a in the liver of Mdr2KO mice compared to FVBN control mice, and found that both of these genes were downregulated in the cholestatic mice. The cellular distribution of Ghr and its receptor GHS-R1a in various types of cells in the liver, was also studied, suggesting that Ghr and its receptor were expressed more in cholangiocytes and hepatocytes than in HSC, in FVBN mice, and both Ghr and GHS-R1a were downregulated in these cells of Mdr2KO mice. These results indicate that in addition to Ghr being decreased in the liver of cholestatic mice, GHS-R1a was also downregulated in these mice, impairing Ghr function in the liver. Treatment of cholestatic mice with Ghr increased the expression of GHS-R1a, suggesting a positive feedback loop of GHS-R1a regulation by Ghr. When Ghr was exogenously administered to cholestatic mice, there was a remarkable improvement of the IBDM and fibrosis markers.

The influence of DG and Ghr on proinflammatory and profibrogenic genes in Mdr2KO mice vs FVBN control mice was also addressed in our study. Ghr significantly reduced the liver levels of cytokines such as CCL2, IL-1β and IL-6, as well as profibrogenic genes including TGFβ, PDGFα, CTGF. Interestingly, DG was also effective in attenuating the expression of the tested profibrogenic genes, especially CTGF. However, a significant effect in terms of expression of ECM proteins such as FN1, Col 1A1, integrin αvβ6, was observed only for Ghr in its acylated form.

We focused on a possible effect of Ghr on cholangiocytes in particular, based on the observation that the IBDM was significantly reduced in cholestatic mice when treated with Ghr. Using mouse cholangiocytes in culture, we demonstrated that Ghr-activated GHS-R1 receptor initiated a signaling pathway conducive to FOXO1 activation and negative regulation of cell proliferation. Thus, our results indicate that Ghr treatment of cholangiocytes caused activation of AMPK via a Ca^2+^-dependent signaling, since BAPTA, a Ca^2+^ chelator, prevented this process. Moreover, Ghr induced FOXO1 nuclear translocation, FOXO1 activation, and a significant decrease in proliferation of cholangiocytes. Both BAPTA and an inhibitor of AMPK activity, dorso morphine, had a negative effect on Ghr-induced nuclear localization and activation of FOXO1. Several studies on FOXO1 roles in the regulation of cell cycle progression have found that FOXO1 has a growth-inhibitory effect through apoptosis or cell cycle arrest^47,48^. Thus, FOXO1 is a member of FOXO transcription factors that induce cell cycle arrest at G1 phase by modulation of CDK (cycle-dependent kinase) inhibitors p27KIP1 and p21 WAF, as well as cyclins D1 and D2^36,49–51^. The subcellular localization of FOXO1 can be influenced by post-translational modifications including acetylation by sirtuins, or serine/threonine phosphorylation by nuclear or cytoplasmic protein kinases. FOXO1 was demonstrated to be involved in progranulin (PGRN)-induced proliferation of cholangiocytes, where PGRN decreased sirtuin 1 expression and increased acetylation of FOXO1 resulting in the cytoplasmic accumulation of FOXO1^52,53^. In cholangiocarcinoma cells, IL-6-dependent activation of PGRN increased cell proliferation by a mechanism involving protein kinase B (Akt) phosphorylation followed by nuclear exclusion of FOXO1^54^. In this study, we demonstrate that unlike PGRN, Ghr induces the activation of FOXO1 and its translocation from cytoplasm into nuclei, decreasing the cell proliferation of cholangiocytes in vitro. We also showed that GHS-R1a knockdown with siRNA, impaired Ghr signaling in cholangiocytes, suggesting that Ghr effects on AMPK/FOXO1-mediated control of cholangiocyte proliferation is mediated by this receptor.

In regard to Ghr-induced activation of AMPK via GHS-R1a, it is known that GHS-R1a, once activated, acts as a G-protein coupled receptor upon plasma membrane phospholipases which then produce inositol triphosphate (IP3), a secondary messenger to endoplasmic reticulum causing the release of Ca^2+^ into the cytoplasm. The increase in Ca^2+^ stimulates calmodulin-dependent protein kinase kinase (CaMKK) to phosphorylate and activate AMPK^55–57^. AMPK is a cellular energy sensor and its beneficial role in ameliorating hepatic fibrosis has been extensively reviewed^58^. However, the role of AMPK as related to Ghr and its receptor in cholangiocytes is newly described in this work. Previously published data show that AMPK becomes activated in response to various stimuli including low levels of ATP, increased metabolic and oxidative stress, and promotes expression of genes related to energy production and resistance to cellular stress, in a FOXO-dependent manner^59,60^. In cholestasis, cholangiocytes undergo a process of activation also known as ductular reaction, in which the cell proliferation rate is abnormally increased^61–63^, and FOXO1, a member of FOXO family of transcription factors with roles in cell fate regulation, is expressed in cholangiocytes. Our data suggest that Ghr acts via GHS-R1a and modulates the nuclear vs cytoplasmic distribution of FOXO1, causing a reduction in cholangiocyte proliferation.

In conclusion, Ghr levels in serum, liver and stomach of Mdr2KO mice were significantly lower compared to FVBN control mice. Exogenously administered Ghr reduced serum liver enzyme levels, biliary hyperplasia and hepatic fibrosis in the Mdr2KO mouse model of hepatic cholestasis. In vitro experiments using mouse cholangiocytes demonstrated that Ghr induced activation of AMPK and FOXO1 and reduced cell proliferation. All these results suggest that Ghr and its receptor GHS-R1a have relevant roles in modulating bile duct proliferation and liver fibrogenesis in the context of cholestasis.

## Methods

### Chemicals, kits, antibodies, tissue culture media

All chemicals were purchased from Millipore-Sigma (Burlington, MA) unless otherwise stated, and were of the highest grade available. Ghrelin Enzyme Immunoassay (EIA) kit was purchased from Phoenix Pharmaceuticals, Inc (Burlingame, CA). RNeasy kit for isolation of RNA from cells and tissue was from Qiagen (Frederik, MD). Recombinant rat Ghr, DG, BAPTA (Ca^2+^ chelator, inhibitor of AMPK phosphorylation) and DM (inhibitor of AMPK activity) were purchased from Tocris Bioscience (Minneapolis, MN). Hydroxyproline assay kit was purchased from Millipore Sigma (St. Louis, MO). Quantikine ELISA for CCL2 was from R&D Systems (Minneapolis, MN). TGFβ ELISA kit was from InVitrogen (Carlsbad, CA). The Apoptosis/Necrosis Detection kit was from Abcam (Cambridge, MA). Sirius Red Staining kit was from IHC World (Ellicott City, MD). Hematoxylin and VectaStain kits for IHC staining were from Vector Laboratories (Burlingame, CA). For IHC, immunofluorescence (IF) and LCM assays, the following antibodies from Abcam (Cambridge, MA) were used: Ghr, GHS-R1a, CK19, CK7, CK8, Alb, desmin, αSMA. We also used antibody to integrin αvβ6 from Bioss Antibodies (Woburn, MA), anti-IDU antibody (B44) from BD Biosciences (San Jose, CA). PCNA antibody was purchased from Santa Cruz Biotechnology (Dallas, TX). Culture media (MEM) and the supplements, i.e. fetal bovine serum (FBS) and penicillin/streptomycin (P/S), were from Gibco BRL purchased through ThermoFisher Scientific (Waltham, MA).

### Animal experiments

FVB/NJ (FVBN) and Mdr2-knockout (Mdr2KO, FVBN background) mice were purchased from The Jackson Laboratory (Bar Harbor, ME) and maintained in a temperature-controlled environment at 20–22 °C with a 12:12 h light–dark cycle, having free access to food and drinking water. The JAX ID’s of Mdr2KO and FVBN mice are FVB.129P2-*Abcb4*^*tm1Bor*^/J #002539, and FVB/NJ stock #001800. The Mdr2KO and FVBN mice were genotyped and confirmed to have the respective genes as expected (Supplemental Fig. 3). For genotyping we followed the protocol from Jackson Laboratory (https://www.jax.org) for Mdr2KO mice, and used Clontech polymerase kit from Fisher Scientific (Waltham, MA). The primers were purchased from Integrated DNA Technologies (Coralville, IA) and had the following sequences: (i) for mutant reverse, 5′-GCT ACT TCC ATT TGT CAC GTC C-3′; (ii) for both FVBN and Mdr2KO mice, forward primer: 5′-TGG GAA GAG TGG AGA AAT CG-3′; (iii) for wild type reverse: 5′-TGA AGA CAT CGG TGT TCA GAG-3’.

All animal procedures were performed in accord with approval of the Institutional Animal Care and Use Committee of the University of Texas at Austin. In experiments designed to measure the effect of Ghr on liver fibrosis in Mdr2KO mice, two month old male and female Mdr2KO mice were used with age-matched FVBN mice as negative controls. The mice were administered vehicle (saline solution), DG or Ghr using osmotic minipumps from Alzet Osmotic Pumps (Cupertino, CA), and DG/Ghr were administered at a rate of 100 µg/kg/day for 14 days, after which the mice were euthanized for blood, stomach and liver sample collection. The 100 µg/kg/day dose was chosen based on previously reported studies in which DG was used to inhibit cancer cell growth in mice, without affecting hepatic functions^64^.

### Assessment of mRNA expression

Assessment of gene expression at the mRNA level in liver tissue or cultured cells was performed by real time quantitative PCR (RT-qPCR) for Ghr, GHS-R1, CK19, PCNA, desmin, αSMA, Col1A1, MMP2, TIMP1, integrin β6, integrin αv, FN1, TGFβ, PDGFα, CTGF, CCL2, IL-1β, IL-6. Fold changes in gene expression were normalized to glyceraldehyde 3-phosphate dehydrogenase (GAPDH). Total RNA was isolated by using RNeasy kit, followed by cDNA synthesis with iScript kit from Bio-Rad Life Sciences (Hercules, CA), and RT-qPCR using iTaq Universal SYBR-Green Supermix from the same company. RT^2^ qPCR Primer Assays were purchased from Qiagen (Frederik, MD). A list of all primers used, is presented in supplemental Table 1. AriaMx Real-Time PCR system thermal cycler from Agilent Technologies (Santa Clara, CA) was used for running RT-qPCR. The data was analyzed as previously described^65^.

### Assessment of biliary hyperplasia and liver fibrosis in Mdr2KO and FVBN mice

The IBDM was assessed by immunohistochemistry (IHC) for CK19 to estimate biliary hyperplasia. The hepatic fibrosis markers desmin, αSMA and integrin αvβ6 were assayed by IHC of liver tissue from mice treated with vehicle, DG or Ghr. Liver tissue sections (4 µm thick) were immunolabeled with primary antibodies specific to proteins of interest, and then processed for staining with VectaStain kits (Burlingame, CA). Solutions of 2–5 µg/mL primary antibodies were used. The IHC slides were scanned with a Leica SCN400 scanner at 20 × magnification, followed by screenshots at 10 × magnification, and image analysis with ImageJ software version 1.41, downloaded from the NIH website (https://imagej.nih.gov). For all samples and controls, the percent areas of stained pixels were calculated and compared for significant differences. Liver samples were also assayed by using Sirius Red specific staining of collagens I and III which are increased in hepatic fibrosis, with the kit from IHC World (Ellicott City, MD).

### Assessment of serum Ghr, liver enzymes, cytokines and growth factors

Ghrelin was assessed in serum of Mdr2KO and FVBN mice, using an ELISA kit purchased from Phoenix Pharmaceuticals, Inc (Burlingame, CA), according to the manufacturer’s instructions. Serum markers for liver function, ALT and AST, from mice treated with vehicle, Ghr or DG were assessed using the IDEXX Catalyst One analyzer from IDEXX Laboratories (Westbrook, ME). Proinflammatory cytokine CCL2 and profibrogenic growth factor TGFβ1 were assessed using ELISA kits from ThermoFisher (Waltham, MA).

### Assessment of AMPK and FOXO1 activation in mouse cholangiocytes

Cholangiocytes were treated with vehicle, Ghr, Ghr and BAPTA or Ghr and DM for 15 min to 2 h and then processed for ELISA for p-AMPK with the PathScan Phospho-AMPK (Thr172) ELISA kit from Cell Signaling Technology Inc. (Danvers, MA), and TransAM FKHR kit from Active Motif (Carlsbad, CA). Prior to the ELISA assay for FKHR or FOXO1 activity, the cell nuclei were isolated using a Nuclear Extract kit from Active Motif (Carlsbad, CA).

Cell proliferation of cholangiocytes treated with Ghr in the absence or presence of BAPTA or DM was assessed using the MTS kit from Abcam (Cambridge, MA), according to manufacturer’s instructions. Incorporation of IDU (5-Iodo-2′-deoxyuridine from Millipore/Sigma, Saint Louis, MO) into DNA in proliferative cholangiocytes was also performed using BrdU/IdU-specific antibody from BD Biosciences (San Jose, CA). Briefly, the cells were incubated with culture medium containing 25 µM IDU for 2 h at 37 °C under 5% CO_2_, then washed with PBS, fixated with 4% paraformaldehyde, permeabilized with 0.5% Triton in PBS (PBS-Triton), followed by treatments with 1 N and 2 N HCl for 10 min each on ice, methanol/acetic acid (3:1 vol/vol) for 10 min at room temperature and washes with PBS-Triton. The cells were further immunolabeled with BrdU/IDU antibody (5 µg/mL) at 4 °C overnight followed by secondary antibody-conjugated with fluorescent dye for 1 h at room temperature. The proliferative cells were counted on confocal images, and the results were expressed in % cells that had IDU incorporated in their nuclei.

### Assessment of Ghr and GHS-R1a expression in the liver by LCM

Frozen sections of liver (8 µm thick) from two month old male and female FVBN and Mdr2KO mice were processed for IF by blocking of nonspecific binding with 4% bovine serum albumin (BSA) in phosphate buffer saline (PBS) supplemented with 0.5% Tween 20 (PBST), followed by two hour incubation with 2–5 µg/mL primary antibody in PBST/BSA at 4 °C, and subsequent labeling with Alexa Fluor 488-conjugated secondary antibody incubation for 1 h at room temperature. The liver sections were labeled with antibodies specific to markers of cholangiocytes, hepatocytes and HSC. Subsequently, a Leica LMD7000 microdissection system (Temple Health & Bioscience District, Temple TX) was used to isolate the specific liver cells. The RNA isolation from batches of 500–1000 cells, was achieved using Arcturus PicoPure Frozen RNA Isolation kit from Thermo Fisher Scientific (Waltham, MA). The expression of Ghr, GHS-R1a and β-actin was then assessed by the same procedure described for RT-qPCR.

### Assessment of Ghr distribution in different types of liver cells by confocal microscopy

The presence of Ghr in cholangiocytes, HSC and hepatocytes, was detected by double fluorescent labeling of frozen liver sections with a mix of antibodies specific to Ghr and one of the three cell markers, i.e. CK19, CK8 and desmin. The overlay of red fluorescence-labeled Ghr with the green fluorescence-labeled cells was observed by using a confocal laser scanning system from Leica Microsystems Inc. (Buffalo Grove, IL).

### Assessment of apoptosis and necrosis in liver from FVBN and Mdr2KO mice

We used an Apoptosis/Necrosis detection kit from Abcam (catalog number ab176749) and followed the manufacturer’s instructions with a few adjustments for frozen sections. Thus, frozen sections of livers from Mdr2KO and FVBN mice were washed briefly with PBS, fixed with 4% paraformaldehyde for 5 min at room temperature and incubated with 1 × Apopxin Green Indicator to stain phosphatidylserine which is exposed by apoptotic cells. The liver sections were then washed briefly with PBS followed by incubation with 1 × 7-AAD (7-aminoactinomycin D), a membrane impermeable dye which labels nuclei of damaged cells. Cells labeled by 7-AAD appear in red fluorescence in confocal microscopy. In the end the sections were mounted in Prolong Gold antifade mountant with DAPI from InVitrogen (Carlsbad, CA).

### Knockdown of GHS-R1a mRNA in mouse cholangiocytes

Mouse cholangiocytes were plated in six-well plates (for ELISA assays) or in chamber slides (for IF assays) at 30–40% confluency and transfected the same day using Lipofectamine 2000 according to manufacturer’s instructions. The GHS-R1a and negative control siRNAs were purchased from Ambion through Thermofisher (Waltham, MA). Validation for GHS-R1a knockdown was performed in cholangiocytes 48 h after the siRNA transfection, using RT^2^ qPCR Primer Assays for GHS-R1a purchased from Qiagen (Frederik, MD). Also, at 48 h after the transfection, the cells were treated with vehicle or Ghr for 30 min, and then processed for ELISA or IF and confocal microscopy. Cell proliferation of transfected cells was also assessed using the MTS kit from Abcam. In this experiment the cells were plated in 96-well plate with transfection reagents, incubated for 48 h, followed by treatments with vehicle or Ghr in the presence of MTS reagent for 4 h.

### Statistics

Quantifications by RT-qPCR, ELISA and image analysis were analyzed by calculating the average and standard error of the mean (SEM) of at least three replicates for each group of tested animals. The number of animals (n) used for each treatment or control group was 4–5, as specified in the Results section for each experiment. The statistical difference was calculated between two groups by using the Student’s T-test, and was considered significant for p values less than 0.05. When multiple groups of animals were compared, a two-way ANOVA was used followed by an appropriate post-hoc test using GraphPad Prism software (San Diego, CA).

## Supplementary information

Supplementary Information.

## References

[1] Al Massadi, O., Lopez, M., Ferno, J., Dieguez, C. & Nogueiras, R. What is the real relevance of endogenous ghrelin? *Peptides* **70**, 1–6 (2015). 10.1016/j.peptides.2015.04.027 26003396

[2] Lim CT, Kola B, Korbonits M (2011). The ghrelin/GOAT/GHS-R system and energy metabolism. Rev. Endocr. Metab. Disord..

[3] St-Pierre DH, Wang L, Tache Y (2003). Ghrelin: A novel player in the gut-brain regulation of growth hormone and energy balance. News Physiol. Sci..

[4] Takahashi H (2006). Ghrelin enhances glucose-induced insulin secretion in scheduled meal-fed sheep. J. Endocrinol..

[5] Meier U, Gressner AM (2004). Endocrine regulation of energy metabolism: Review of pathobiochemical and clinical chemical aspects of leptin, ghrelin, adiponectin, and resistin. Clin. Chem..

[6] Muller TD (2015). Ghrelin. Mol. Metab..

[7] Callahan HS (2004). Postprandial suppression of plasma ghrelin level is proportional to ingested caloric load but does not predict intermeal interval in humans. J. Clin. Endocrinol. Metab..

[8] Cummings DE, Frayo RS, Marmonier C, Aubert R, Chapelot D (2004). Plasma ghrelin levels and hunger scores in humans initiating meals voluntarily without time- and food-related cues. Am. J. Physiol. Endocrinol. Metab..

[9] Howard AD (1996). A receptor in pituitary and hypothalamus that functions in growth hormone release. Science.

[10] Liu B, Garcia EA, Korbonits M (2011). Genetic studies on the ghrelin, growth hormone secretagogue receptor (GHSR) and ghrelin O-acyl transferase (GOAT) genes. Peptides.

[11] Tong J (2013). The pharmacokinetics of acyl, des-acyl, and total ghrelin in healthy human subjects. Eur. J. Endocrinol..

[12] Ariyasu H (2005). Transgenic mice overexpressing des-acyl ghrelin show small phenotype. Endocrinology.

[13] Yang J, Brown MS, Liang G, Grishin NV, Goldstein JL (2008). Identification of the acyltransferase that octanoylates ghrelin, an appetite-stimulating peptide hormone. Cell.

[14] Gutierrez JA (2008). Ghrelin octanoylation mediated by an orphan lipid transferase. Proc. Natl. Acad. Sci. U S A.

[15] Banks WA, Burney BO, Robinson SM (2008). Effects of triglycerides, obesity, and starvation on ghrelin transport across the blood-brain barrier. Peptides.

[16] De Vriese C (2004). Ghrelin degradation by serum and tissue homogenates: Identification of the cleavage sites. Endocrinology.

[17] Yoshimoto A (2002). Plasma ghrelin and desacyl ghrelin concentrations in renal failure. J. Am. Soc. Nephrol..

[18] Dornelles CT (2013). Ghrelin, leptin and insulin in cirrhotic children and adolescents: Relationship with cirrhosis severity and nutritional status. Regul. Pept..

[19] Tacke F (2003). Ghrelin in chronic liver disease. J. Hepatol..

[20] Marchesini G (2004). Plasma ghrelin concentrations, food intake, and anorexia in liver failure. J. Clin. Endocrinol. Metab..

[21] Takahashi H, Kato A, Onodera K, Suzuki K (2006). Fasting plasma ghrelin levels reflect malnutrition state in patients with liver cirrhosis. Hepatol. Res..

[22] Breidert M, Zimmermann TF, Schneider R, Ehninger G, Brabant G (2004). Ghrelin/leptin-imbalance in patients with primary biliary cirrhosis. Exp. Clin. Endocrinol. Diabetes.

[23] Moreno M (2010). Ghrelin attenuates hepatocellular injury and liver fibrogenesis in rodents and influences fibrosis progression in humans. Hepatology.

[24] Iseri SO (2008). Ghrelin alleviates biliary obstruction-induced chronic hepatic injury in rats. Regul. Pept..

[25] Smit JJ (1993). Homozygous disruption of the murine mdr2 P-glycoprotein gene leads to a complete absence of phospholipid from bile and to liver disease. Cell.

[26] Lammert F (2004). Spontaneous cholecysto- and hepatolithiasis in Mdr2-/- mice: a model for low phospholipid-associated cholelithiasis. Hepatology.

[27] Katzenellenbogen M (2007). Molecular mechanisms of liver carcinogenesis in the mdr2-knockout mice. Mol. Cancer Res..

[28] Trauner M, Fickert P, Wagner M (2007). MDR3 (ABCB4) defects: A paradigm for the genetics of adult cholestatic syndromes. Semin. Liver Dis..

[29] Rosmorduc O, Hermelin B, Poupon R (2001). MDR3 gene defect in adults with symptomatic intrahepatic and gallbladder cholesterol cholelithiasis. Gastroenterology.

[30] Jacquemin E (2001). The wide spectrum of multidrug resistance 3 deficiency: From neonatal cholestasis to cirrhosis of adulthood. Gastroenterology.

[31] Petrescu, A.D.*, et al.* Glucocorticoids cause gender-dependent reversal of hepatic fibrosis in the MDR2-knockout mouse model. *Int. J. Mol. Sci.* **18 **(2017). 10.3390/ijms18112389 PMC5713358 29125588

[32] Petrescu AD (2020). Coordinated targeting of galanin receptors on cholangiocytes and hepatic stellate cells ameliorates liver fibrosis in multidrug resistance protein 2 knockout mice. Am. J. Pathol..

[33] Meng F (2018). Ursodeoxycholate inhibits mast cell activation and reverses biliary injury and fibrosis in Mdr2(-/-) mice and human primary sclerosing cholangitis. Lab Invest..

[34] Kennedy L (2018). Blocking H1/H2 histamine receptors inhibits damage/fibrosis in Mdr2(-/-) mice and human cholangiocarcinoma tumorigenesis. Hepatology.

[35] Jones H (2016). Inhibition of mast cell-secreted histamine decreases biliary proliferation and fibrosis in primary sclerosing cholangitis Mdr2(-/-) mice. Hepatology.

[36] Schmidt M (2002). Cell cycle inhibition by FoxO forkhead transcription factors involves downregulation of cyclin D. Mol. Cell Biol..

[37] Frescas D, Valenti L, Accili D (2005). Nuclear trapping of the forkhead transcription factor FoxO1 via Sirt-dependent deacetylation promotes expression of glucogenetic genes. J. Biol. Chem..

[38] Scerif M, Goldstone AP, Korbonits M (2011). Ghrelin in obesity and endocrine diseases. Mol. Cell Endocrinol..

[39] Sun Y (2014). Ghrelin suppresses Purkinje neuron P-type Ca(2+) channels via growth hormone secretagogue type 1a receptor, the betagamma subunits of Go-protein, and protein kinase a pathway. Cell Signal.

[40] Andrews ZB (2011). The extra-hypothalamic actions of ghrelin on neuronal function. Trends Neurosci..

[41] Castaneda TR, Tong J, Datta R, Culler M, Tschop MH (2010). Ghrelin in the regulation of body weight and metabolism. Front. Neuroendocrinol..

[42] Gonzalez-Rey E, Chorny A, Delgado M (2006). Therapeutic action of ghrelin in a mouse model of colitis. Gastroenterology.

[43] Granata R (2007). Acylated and unacylated ghrelin promote proliferation and inhibit apoptosis of pancreatic beta-cells and human islets: involvement of 3',5'-cyclic adenosine monophosphate/protein kinase A, extracellular signal-regulated kinase 1/2, and phosphatidyl inositol 3-Kinase/Akt signaling. Endocrinology.

[44] Li L (2006). Cardioprotective effects of ghrelin and des-octanoyl ghrelin on myocardial injury induced by isoproterenol in rats. Acta Pharmacol. Sin..

[45] Ceranowicz, P.*, et al.* Essential role of growth hormone and IGF-1 in therapeutic effect of ghrelin in the course of acetic acid-induced colitis. *Int. J. Mol. Sci.* **18 **(2017). 10.3390/ijms18061118 PMC5485942 28538694

[46] Kabil NN, Seddiek HA, Yassin NA, Gamal-Eldin MM (2014). Effect of ghrelin on chronic liver injury and fibrogenesis in male rats: Possible role of nitric oxide. Peptides.

[47] Dijkers PF, Medema RH, Lammers JW, Koenderman L, Coffer PJ (2000). Expression of the pro-apoptotic Bcl-2 family member Bim is regulated by the forkhead transcription factor FKHR-L1. Curr. Biol..

[48] Xing YQ (2018). The regulation of FOXO1 and its role in disease progression. Life Sci..

[49] Kops GJ (2002). Control of cell cycle exit and entry by protein kinase B-regulated forkhead transcription factors. Mol. Cell Biol..

[50] Dijkers PF (2000). Forkhead transcription factor FKHR-L1 modulates cytokine-dependent transcriptional regulation of p27(KIP1). Mol. Cell Biol..

[51] Medema RH, Kops GJ, Bos JL, Burgering BM (2000). AFX-like Forkhead transcription factors mediate cell-cycle regulation by Ras and PKB through p27kip1. Nature.

[52] Frampton G (2012). The novel growth factor, progranulin, stimulates mouse cholangiocyte proliferation via sirtuin-1-mediated inactivation of FOXO1. Am. J. Physiol. Gastrointest. Liver Physiol..

[53] Demorrow S (2013). Progranulin: A novel regulator of gastrointestinal cancer progression. Transl. Gastrointest. Cancer.

[54] Frampton G (2012). Interleukin-6-driven progranulin expression increases cholangiocarcinoma growth by an Akt-dependent mechanism. Gut.

[55] Carling D (2017). AMPK signalling in health and disease. Curr. Opin. Cell Biol..

[56] Hardie DG, Ross FA, Hawley SA (2012). AMPK: A nutrient and energy sensor that maintains energy homeostasis. Nat. Rev. Mol. Cell Biol..

[57] Hurley RL (2005). The Ca2+/calmodulin-dependent protein kinase kinases are AMP-activated protein kinase kinases. J. Biol. Chem..

[58] Liang Z (2017). AMPK: A novel target for treating hepatic fibrosis. Oncotarget.

[59] Greer EL, Banko MR, Brunet A (2009). AMP-activated protein kinase and FoxO transcription factors in dietary restriction-induced longevity. Ann. N. Y. Acad. Sci..

[60] Yun H (2014). AMP-activated protein kinase mediates the antioxidant effects of resveratrol through regulation of the transcription factor FoxO1. FEBS J..

[61] McMillin M, Frampton G, Grant S, DeMorrow S (2017). The neuropeptide galanin is up-regulated during cholestasis and contributes to cholangiocyte proliferation. Am. J. Pathol..

[62] Quinn M (2012). Suppression of the HPA axis during extrahepatic biliary obstruction induces cholangiocyte proliferation in the rat. Am. J. Physiol. Gastrointest. Liver Physiol..

[63] Hall C (2017). Regulators of cholangiocyte proliferation. Gene Expr..

[64] Maugham ML (2018). No effect of unacylated ghrelin administration on subcutaneous PC3 xenograft growth or metabolic parameters in a Rag1-/- mouse model of metabolic dysfunction. PLoS ONE.

[65] Livak KJ, Schmittgen TD (2001). Analysis of relative gene expression data using real-time quantitative PCR and the 2(-Delta Delta C(T)) method. Methods.

