# Supplementary material for: Ghrelin reverses ductular reaction and hepatic fibrosis in a rodent model of cholestasis
Source: Sci Rep. 2020 Sep 29;10:16024. doi: 10.1038/s41598-020-72681-5 (PMC7525536; doi:10.1038/s41598-020-72681-5)
Supplement: Supplementary file 1 — Supplementary Information. [file 41598_2020_72681_MOESM1_ESM.pdf]

Supplementary information

Ghrelin reverses ductular reaction and hepatic fibrosis in a rodent model of cholestasis

Anca D. Petrescu[1,2] , Stephanie Grant[1,2], Elaina Williams[1,2], Gabriel Frampton[3], Evan H. Reinhart[4], Amy Nguyen[5], Suyeon An[1,2], Matthew McMillin[1,3], Sharon DeMorrow [1,2,3]*

[1] Central Texas Veterans Health Care System, Temple 76504, Texas, USA
[2] Division of Pharmacology and Toxicology, College of Pharmacy, The University of Texas at Austin, Austin, Texas 78712, USA
[3] Department of Internal Medicine, Dell Medical School, The University of Texas at Austin, Austin, Texas 78701, USA
[4] Department of Internal Medicine, Baylor Scott & White Health, Temple, Texas 76502, USA
[5] University of Mary Hardin-Baylor, Belton, Texas 76513, USA

Supplemental Table 1. Serum albumin (ALB) and total bilirubin (TBil) in Mdr2KO mice compared to FVBN control mice treated with vehicle, DG or Ghr. The serum was tested for ALB and Tbil using an IDEXX system, as described in the Methods.

**Type of mice and treatments**	**ALB (µmol/L)**	**TBil (µg/dL)**
Male FVBN + vehicle	5.87 ± 0.97	0.91 ± 0.06
Male Mdr2KO + vehicle	10.55 ± 3.14	0.76 ± 0.04
Female FVBN + vehicle	4.92 ± 2.57	0.88 ± 0.10
Female Mdr2KO + vehicle	13.82 ± 4.95	0.63 ± 0.19
Male FVBN + DG	5.53 ± 0.22	0.90 ± 0.03
Male Mdr2KO + DG	6.30 ± 0.48	0.83 ± 0.01
Female FVBN + DG	4.91 ± 0.87	0.92 ± 0.04
Female Mdr2KO + DG	5.89 ± 1.05	0.85 ± 0.06
Male FVBN + Ghr	4.96 ± 0.10	0.94 ±0.02
Male Mdr2KO + Ghr	6.70 ± 0.46	0.85 ± 0.02
Female FVBN + Ghr	5.74 ± 0.09	0.95 ± 0.04
Female Mdr2KO + Ghr	5.23 ± 0.12	0.83 ± 0.03

Supplemental Table 2. List of primers used in RT-qPCR assays. As mentioned in Methods section, all RT-qPCR assays were run using iTaq Universal SYBR-Green Supermix from Bio-Rad Life Sciences (Hercules, CA) and RT^2 qPCR Primer Assays purchased from Qiagen (Frederik, MD). The following primers have been used:

**Gene**	**Catalog number for primer set**
CTGF	PPM03798B
CCL2	PPM03151G
KRT19	PPM02968A
COL1A1	PPM03845F
DES	PPM25379A
FN1	PPM03786A
GAPDH	PPM02946E
GHRL	PPM31564C
GHSR	PPM05304A
ITGAV	PPM03662D
INTB6	PPM03593A
IL1B	PPM03109F
IL6	PPM03111E
MBOAT1	PPM30343A
MMP2	PPM03642C
PCNA	PPM03456F
PDGFa	PPM03103E
ACTA2	PPM04483A
TGFb1	PPM02991B
TIMP1	PPM03693F

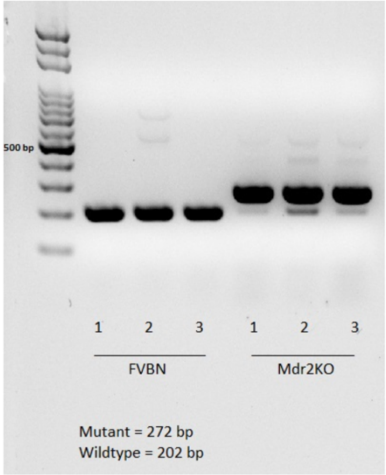

Supplemental Figure 1. Genotyping of Mdr2Ko mice vs FVBN control mice. Representative image of DNA gel where specific DNA markers of Mdr2KO (or Mdr2$^{-/-}$) mice and of wild type (or Mdr2$^{+/+}$) mice, were detected as a 272 base pair band, and a 202 base pairs band, respectively. The DNA bands were obtained as described in the Methods section.

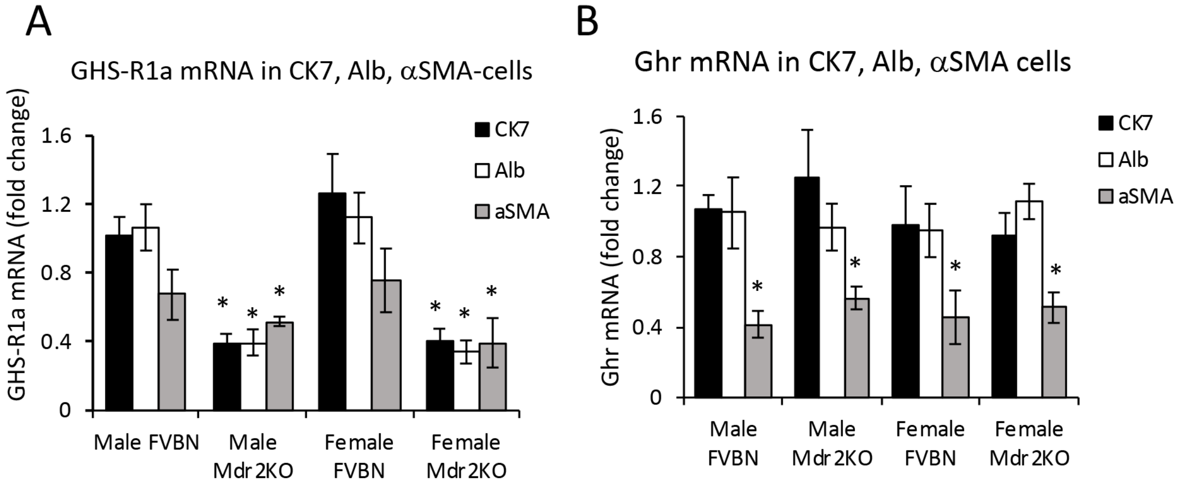

Supplemental Figure 2. GHS-R1a and Ghr mRNA expression in different types of cells in liver tissue from Mdr2KO mice and FVBN controls. Frozen sections of the livers were immunolabeled for CK7 (cholangiocytes), albumin (ALB, hepatocytes) or αSMA (activated HSC) and laser capture microdissection (LCM) was used to selectively dissect specific cells as described under Methods. RT-qPCR was then used to quantify the relative expression of GHS-R1a and Ghr vs GAPDH in the dissected cells. A, results for GHSR-1a. B, results for Ghr. and CK7(B) for cholangiocytes, CK8 (A) and albumin (Alb, B) for hepatocytes and desmin (A) or αSMA(B) for HSC. Number of animals for each type of treatment, 4. Number of sample replicates for RT-qPCR assay, 3. $p<0.05$. *, Mdr2KO vs FVBN mice.

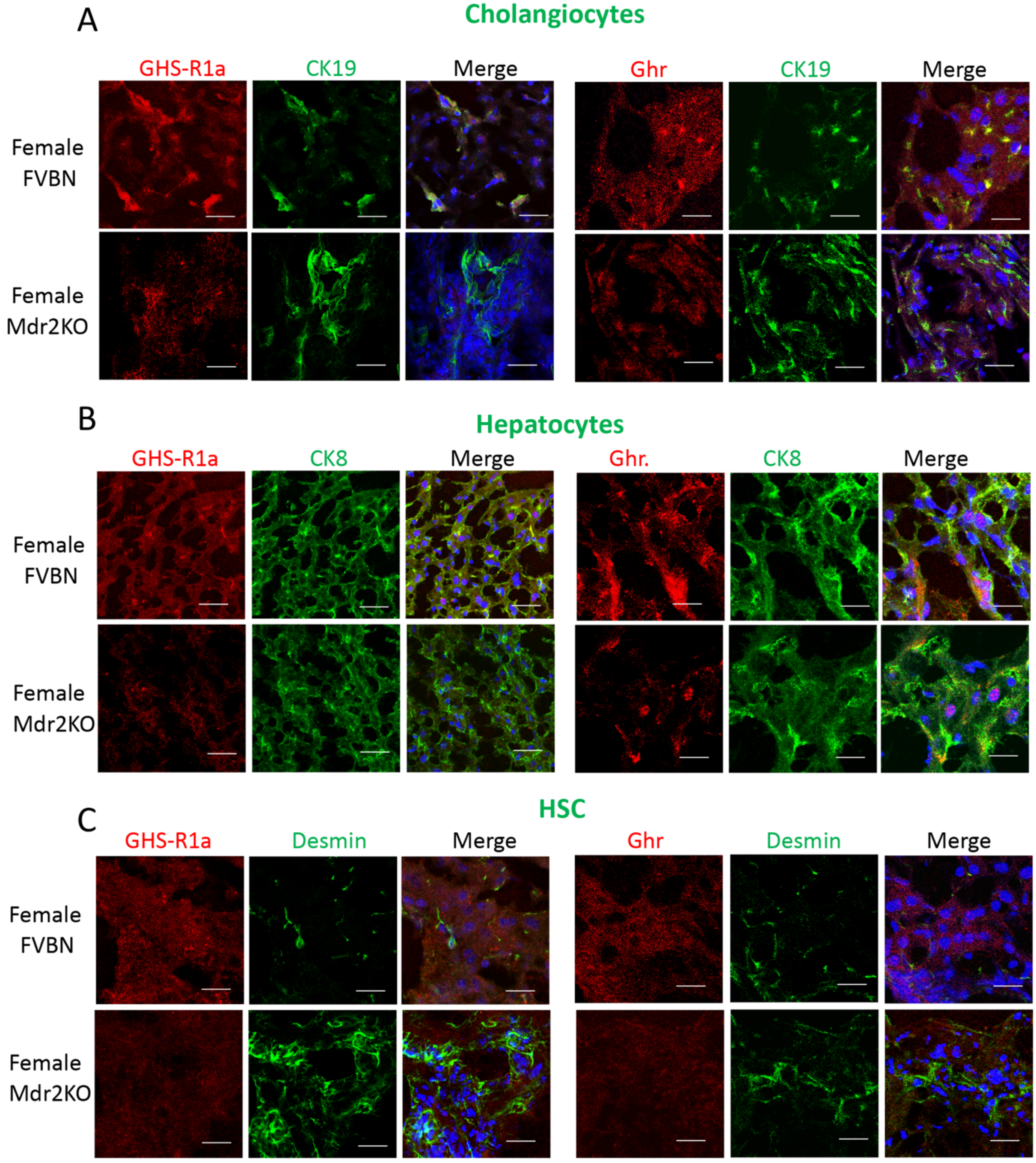

Supplemental Figure 3. Distribution of GHS-R1a and Ghr in different types of cells in liver samples of Mdr2KOmice and FVBN controls. Confocal microscopy was used to assess colocalization of GHS-R1a or Ghr (immuno-detected in red) and CK19, CK8 or desmin (immuno-detected in green) in liver sections, as described under Methods. A, Images of GHS-R1a (left group) and Ghr (right group) colocalizing with CK19 marker of cholangiocytes. B, Images of GHS-R1a (left) and Ghr (right) colocalizing with CK8 marker of hepatocytes. C, Images of GHS-R1a (left) and Ghr (right) colocalizing with desmin marker of HSC. Scale bar, 100 μm.

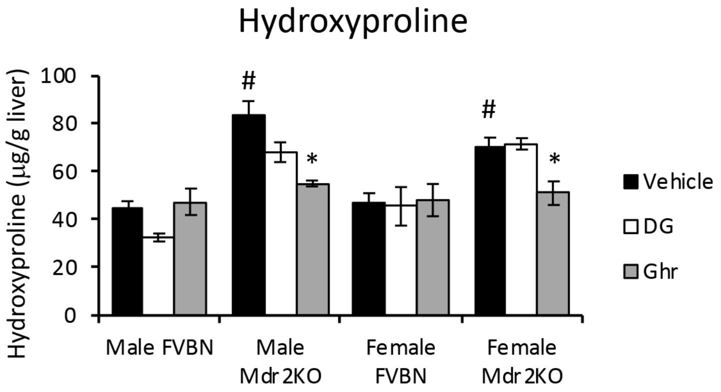

Supplemental Figure 4. Ghr reduces hydroxyproline in Mdr2KO livers. Hydroxyproline is abundant in collagen which is a major component of highly increased ECM, in Mdr2KO mice due to cholestasis-induced fibrogenesis. Hydroxyproline was assayed in livers from Mdr2KO and FVBN mice treated with vehicle, DG or Ghr, using a kit as described under Methods. Number of animals for each type of treatment, 4. Number of sample replicates for assay, 3. $p<0.05$. #, Mdr2KO vs FVBN mice. *, Ghr vs vehicle within the same group of FVBN or Mdr2KO mice.
